# Leveraging Omeprazole PBPK/PD Modeling to Inform Drug–Drug Interactions and Specific Recommendations for Pediatric Labeling

**DOI:** 10.3390/pharmaceutics17030373

**Published:** 2025-03-14

**Authors:** Amira Soliman, Leyanis Rodriguez-Vera, Ana Alarcia-Lacalle, Leandro F. Pippa, Saima Subhani, Viera Lukacova, Jorge Duconge, Natalia V. de Moraes, Valvanera Vozmediano

**Affiliations:** 1 Center for Pharmacometrics and Systems Pharmacology, Department of Pharmaceutics, College of Pharmacy, University of Florida, Orlando, FL 32827, USAlrodriguez@ctifacts.com (L.R.-V.); nataliademoraes@ufl.edu (N.V.d.M.); 2 Department of Pharmacy Practice, Faculty of Pharmacy, Helwan University, Helwan, Cairo 11795, Egypt; 3 Model Informed Development, CTI Laboratories, Covington, KY 41011, USA; 4 Pharmacokinetic, Nanotechnology and Gene Therapy Group (PharmaNanoGene), Faculty of Pharmacy, Centro de Investigación Lascaray Ikergunea, University of the Basque Country UPV/EHU, 01006 Vitoria-Gasteiz, Spain; ana.alarcia@ehu.eus; 5 Bioaraba, Microbiology, Infectious Disease, Antimicrobial Agents, and Gene Therapy, 01009 Vitoria-Gasteiz, Spain; 6 Simulation Plus, Inc., Lancaster, CA 93534, USA; saima.subhani@simulations-plus.com (S.S.); viera.lukacova@simulations-plus.com (V.L.); 7 Department of Pharmaceutical Sciences, School of Pharmacy, University of Puerto Rico, Medical Sciences Campus, San Juan, PR 00936, USA; jorge.duconge@upr.edu

**Keywords:** PBPK-PD, omeprazole, pediatrics, age-physiological changes, developmental changes

## Abstract

**Background/Objectives:** Omeprazole is widely used for managing gastrointestinal disorders like GERD, ulcers, and *H. pylori* infections. However, its use in pediatrics presents challenges due to drug interactions (DDIs), metabolic variability, and safety concerns. Omeprazole’s pharmacokinetics (PK), primarily influenced by CYP2C19 metabolism, is affected by ontogenetic changes in enzyme expression, complicating dosing in children. **Methods**: This study aimed to develop and validate a physiologically based pharmacokinetic (PBPK) model for omeprazole and its metabolites to predict age-related variations in metabolism and response. **Results**: The PBPK model successfully predicted exposure to parent and metabolites in adults and pediatrics, incorporating competitive and mechanism-based inhibition of CYP2C19 and CYP3A4 by omeprazole and its metabolites. By accounting for age-dependent metabolic pathways, the model enabled priori predictions of omeprazole exposure in different age groups. Linking PK to the pharmacodynamics (PD) model, we described the impact of age-related physiological changes on intragastric pH, the primary outcome for proton pump inhibitors efficacy. **Conclusions**: The PBPK-PD model allowed for the virtual testing of dosing scenarios, providing an alternative to clinical studies in pediatrics where traditional DDI studies are challenging. This approach offers valuable insights for accurate dosing recommendations in pediatrics, accounting for age-dependent variability in metabolism, and underscores the potential of PBPK modeling in guiding pediatric drug development.

## 1. Introduction

Proton pump inhibitors (PPIs), especially omeprazole, are commonly used to treat gastrointestinal disorders such as gastroesophageal reflux disease (GERD), ulcers, and *Helicobacter pylori* infections in both adults and children [1]. GERD, affecting about 20% of the global population, is particularly prevalent, but up to 40% of patients may be resistant to PPI treatment [2,3,4,5]. Omeprazole is effective in reducing gastric acid secretion and alleviating GERD symptoms [6]. However, its complex pharmacokinetics (PK) and pharmacodynamics (PD), including drug–drug interactions (DDIs), metabolic variability, and pediatric dosing, pose challenges to achieving consistent therapeutic efficacy and safety across diverse patient populations. These challenges include managing interindividual variability in drug metabolism due to genetic polymorphisms, avoiding adverse DDIs, and determining appropriate dosing strategies for special populations, such as children, to ensure optimal clinical outcomes.

Omeprazole inhibits the H^+^/K^+^-ATPase in the parietal cell, leading to reduced gastric acid secretion [7,8]. Although it is rapidly absorbed and cleared from plasma, its effects last 24–72 h due to covalent binding to proton pumps [8,9]. Omeprazole undergoes extensive first-pass metabolism and is primarily metabolized in the liver via the cytochrome P450 system, with the (*S*)-isomer (esomeprazole) show higher efficacy [9,10]. CYP2C19 and CYP3A4 enzymes play key roles in its metabolism, and genetic differences in CYP2C19 affect PPI exposure and efficacy [11,12]. Omeprazole is also a potent inhibitor of these enzymes, complicating its use in combination with other drugs [13,14,15,16,17].

In pediatric patients, PPI use raises concerns about side effects such as nutrient deficiencies (iron, calcium, vitamin B12) and an increased risk of infections due to elevated intragastric pH [18]. Long-term PPI use in children is associated with adverse outcomes like bone fractures, inflammatory bowel diseases, and asthma, highlighting the need for careful prescription [19]. Additionally, metabolic differences in children may complicate the understanding of dose–exposure–response relationships and DDIs.

Physiologically based pharmacokinetic (PBPK) modeling can help predict drug absorption, distribution, metabolism, and excretion (ADME) across age groups, offering insights into PPI dosing and DDIs in both adults and children [20]. This model is particularly useful for drugs like omeprazole, where PK and PD are not directly correlated due to its covalent binding inhibition of the H^+^/K^+^-ATPase. PBPK can provide a more accurate evaluation of drug effects, especially in pediatric drug development, where metabolic pathways vary by age [21,22].

Drug labels often lack specific DDI recommendations for pediatrics. However, age-dependent changes in the metabolic pathways may impact the effect of DDIs, leading to special dosing needs in pediatrics taking interacting medications. PBPK models can predict these interactions by considering ontogenetic changes in metabolic pathways, providing valuable insights into the potential risks and necessary dosing adjustments. In recent years, regulatory agencies have increasingly endorsed the use of PBPK modeling to predict DDIs, particularly in the context of pediatric drug development [22].

In this study, we aim to (i) develop and validate a PBPK model for omeprazole and its metabolites in adults and extrapolate it to pediatrics, (ii) investigate the impact of CYP enzyme ontogeny on omeprazole disposition, (iii) evaluate age-related changes in omeprazole’s PD, and (iv) assess the impact of DDIs in pediatrics using a scenario where a drug inhibits CYP3A. Using PBPK/PD modeling, we hope to guide safer and more effective pediatric dosing strategies. This approach will also help demonstrate the use of PBPK to address knowledge gaps in DDIs in pediatrics, serving as an example of how pediatric drug development can be tailored to more effectively select drug doses for children, minimizing risks in vulnerable populations.

## 2. Materials and Methods

### 2.1. Software

The software GastroPlus^®^ (version 9.8.2, Simulation Plus Inc., Lancaster, CA, USA) was used for the development and verification of the PBPK model for omeprazole. The ADMET Predictor^®^ Module version 9.5 was used to obtain some physicochemical parameters from the omeprazole compound structure. The DDI module within GastroPlus^®^ was used to predict the competitive inhibition, the mechanism-based inactivation (MBI) for omeprazole and its metabolites. The age-dependent anatomical and physiological parameters of adult humans and pediatrics of different ages used in the PBPK model development have been built into the tool database [23]. Clinical plasma data from the literature was digitized using Graph Grabber version 2.0.2. Pumas^®^ version 2.2.0 (Pumas-AI, Baltimore, MD, USA) [24], an integral package within the Julia programming language, was utilized to develop the PD models. To effectively manage, visualize, and analyze our data, we employed R^®^ version 4.4.1 [25], operated through the user-friendly RStudio [26] interface. Non-compartmental analysis was performed with the R package NonCompart, version 0.7.0.

### 2.2. Clinical PK Data

Clinical plasma concentrations versus time data after single and multiple dose administrations of omeprazole were collected and digitized from literature. A total of 6 clinical studies [27,28,29,30,31,32] with 79 subjects were used to develop and verify the omeprazole PBPK model. A description of these studies is summarized in Table 1.

### 2.3. PBPK Model Development and Verification of Omeprazole in Adults

**Figure 1:**
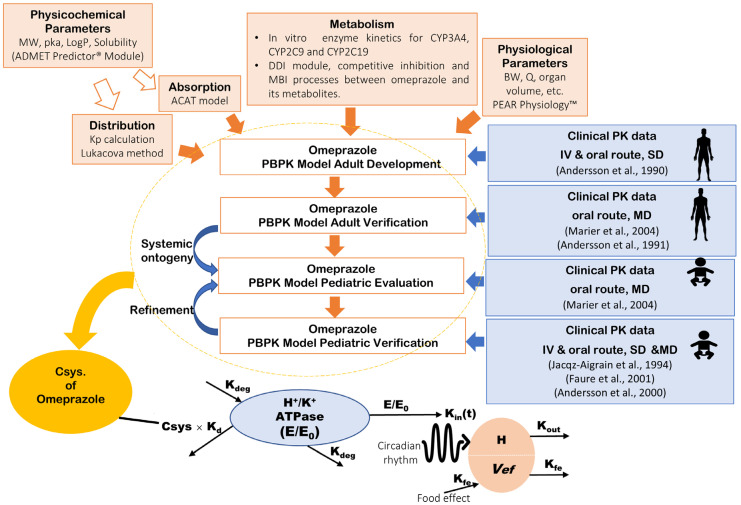
Workflow of the omeprazole PBPK/PD modeling strategy. The top section illustrates the PBPK model development and verification, while the bottom section details the PD model of gastric acid secretion. PBPK: physiologically based pharmacokinetics; MW: molecular weight; logP: partition coefficient; pKa: acid dissociation constant; Kp: tissue plasma partition coefficients; CYP: cytochrome P450; MBI: mechanism-based inactivation; BW: body weight; Q: blood flow rate; SD: single dose; MD: multiple-dose; Csys.: systemic plasma concentration; H^+^/K^+^-ATPase (E/E_0_): relative baseline activity of proton pump predicted as a function of the production or elimination; H: H^+^ concentration in the stomach; V*ef*: food effect; K_d_: Efficacy of omeprazole to irreversibly bind H^+^/K^+^-APTase; K_deg_: Degradation rate constant of H^+^/K^+^ APTase; K_in_(t): Circadian rhythm; K_out_: Elimination rate constant for intra-gastric H^+^ concentration; K_fe_: the first-order rate of volume removal corresponding to gastric emptying. Andersson et al., 1990 [27], Marier et al., 2004 [28], Andersson et al., 1991 [29], Jacqz-Aigrain et al., 1994 [30], Faure et al., 2001 [31], Andersson et al., 2000 [32].

A whole-body PBPK model of omeprazole and its major metabolites (hydroxy-omeprazole and omeprazole sulphone) was built and evaluated using plasma profiles from clinical studies, as depicted in the workflow in Figure 1. The first step in developing the omeprazole PBPK model involved predicting drug-specific parameters using the ADMET Predictor^TM^ Module based on the structure of the omeprazole compound. Concurrently, a comprehensive literature review was conducted to gather experimental physicochemical and biopharmaceutical parameters and all PK/clinical information on omeprazole. Human organ weights, volumes, and blood perfusion rates specific to subjects in each study (sex, age, and body weight) were generated using the GastroPlus^®^ internal Population Estimates for Age-Related (PEAR) Physiology™ Module.

In the PBPK model, tissue/plasma partition coefficients (Kps) for perfusion-limited tissues (liver, lung, spleen, adipose, heart, kidney, reproductive organs, yellow marrow, muscle, brain, skin, red marrow, and rest of the body) were calculated using the Lukacova method [33]. The gut was modeled using the in-built advanced compartment absorption and transit (ACAT) model in GastroPlus^®^. The dissolution rate (z factor) versus time was initially informed by the literature and then manually adjusted to improve the release profile from the delayed-release formulation.

The main metabolites of omeprazole are hydroxy-omeprazole, 5′-O-desmethylomeprazole, omeprazole sulphone and carboxy-omeprazole [27,34]. The relative contribution of CYP2C19, CYP2C9 and CYP3A4 in the metabolism of omeprazole and the subsequent metabolism of the metabolites to secondary metabolites has been previously studied [35,36]. However, the impact of age on enzyme expression and activity has not been thoroughly investigated. To address this gap, the current model incorporates the contributions of CYP2C19, CYP3A4 and CYP2C9 to the formation of hydroxy-omeprazole and omeprazole sulphone and their subsequent metabolism. In this study, the metabolism of omeprazole by CYP2C19, CYP2C9 and CYP3A4 was defined by the Michaelis–Menten constant (Km) and maximum reaction velocity (Vmax) from the in vitro reported values [34]. The expression levels of these metabolic enzymes were based on the in-built expression in GastroPlus^®^ version 9.8.2. The model included the following processes:

1. Liver metabolism of omeprazole by CYP2C19 to form hydroxy–omeprazole and others such as 5′-O-desmethylomeprazole.
2. Liver metabolism of omeprazole by CYP3A4 to form hydroxy–omeprazole, omeprazole sulphone and others such as 5′-O-desmethylomeprazole.
3. Gut metabolism of omeprazole by CYP3A4 to form omeprazole sulphone.
4. Liver metabolism of omeprazole by CYP2C9 to form hydroxy-omeprazole and others such as 5′-O-desmethylomeprazole.

The Vmax for CYP2C19 and CYP3A4 for the parent and metabolites was later optimized using IV and oral plasma concentration data [27] until a reasonable fit to the observed profiles was obtained, maintaining consistent distribution, metabolism and excretion-related inputs unchanged across all models. To build the model, the single-dose IV profile of 40 mg omeprazole reported by Andersson et al., 1990 [27] was simulated, for which the full omeprazole PBPK model was developed with an integrated PBPK model of metabolites. Subsequently, the model was applied to simulate the PK profiles of single oral doses of 40 mg and 90 mg omeprazole reported by Andersson et al., 1990 [27]. Furthermore, competitive inhibition and MBI processes between omeprazole and its metabolites in the CYP2C19 and CYP3A4 enzymes described in the literature [14,15] were also incorporated into the model. All these mechanisms were introduced using the DDI module of GastroPlus^®^. Additionally, the metabolism of the primary metabolites of omeprazole (hydroxy-omeprazole and omeprazole sulphone) was included based on in vitro data [37] for Km and Vmax. Vmax was later optimized to fit the plasma concentration (Cp) versus time profile [27]. Then, the model’s performance was verified after multiple dosing administration of omeprazole [28,29]. The developed adult PBPK model was also externally verified for the contribution of CYP2C19 and CYP2C9 by simulation DDI with fluconazole, a strong CYP2C9 and CYP2C19 inhibitor. Observed [38] and predicted AUC fluconazole/AUC omeprazole were compared.

### 2.4. PBPK Model Development and Verification of Omeprazole in Pediatrics

The verified PBPK model in adults was then extrapolated to pediatrics. Initially, the physiologic information was based on the literature sources [28,30]. Since CYP3A7 is the major fetal form of CYP3A where a developmental switch from CYP3A7 to CYP3A4 expression shortly after birth occurs [39], it was essential to incorporate CYP3A7 metabolism for omeprazole and its metabolites [40]. The Km and Vmax of CYP3A7 were calculated from the Km and Vmax of the CYP3A4, where Km values for CYP3A7 were 5.1 times higher compared to the respective Km values of CYP3A4 for the model substances, Vmax values were 75% lower [41]. Pediatric profiles from Marier et al. [28] were used to evaluate the extrapolated PBPK models from adults to pediatrics.

The developed PBPK model in pediatrics was then verified with the literature-reported PK profiles for pediatric patients [31,32]. Also, different age groups were simulated and compared to the observed AUC (12 to 24 h) reported by Jacqz-Aigrain et al., 1994 [30]. The Du Bois equation was used to estimate the body weight based on the surface area, as the study did not report patients’ body weight [42]. A population simulation (*n* = 25) was performed to incorporate variability in the predictions.

### 2.5. Application of the PBPK Model to Explore the Impact of Age-Dependent Physiological Changes on Omeprazole Exposure and Response

To provide insights into omeprazole metabolism across different age groups, the verified PBPK models of omeprazole and its metabolites were utilized to simulate the administration of a single dose of 10 mg delayed-release enteric-coated tablet to subjects aged 4 months, 6 months, 9 months, 1 year, 2 years, 6 years, 16 years and 32 years. The contribution of the drug-metabolizing enzymes CYP2C19, CYP3A4, CYP2C9 and CYP3A7 to the overall metabolism of omeprazole at different ages was extracted using the PBPK model. Also, the verified PBPK models of omeprazole and its metabolites were utilized to simulate omeprazole exposure in adults and pediatrics of different ages to explore the impact of age-dependent metabolic changes on omeprazole exposure. Dynamic simulations were conducted for five different age groups: adults aged 37 years and pediatric populations aged 6 years, 2 years, 8 months, and 4 months. Initially, single simulations were performed for these five age groups, based on literature data, to verify the model. Subsequently, a population simulation (*n* = 100 subjects) was conducted for these five age groups with omeprazole doses of 20 mg for adults and 0.7 mg/kg for pediatrics [43] once daily for 8 days to ensure steady-state.

The simulated plasma concentration profiles obtained from the PBPK models were then linked to a previously developed and verified mechanism-based PD model [44] to predict intragastric H^+^ concentration using Pumas-AI 2.2.0 (Pumas-AI, Baltimore). The primary goal of PD was to explore whether age-based physiological changes in the GI tract, combined with changes in omeprazole PK, would result in an altered drug response. The indirect response model, which accounts for the irreversible inhibition of H^+^/K^+^ATPase, the circadian rhythm, and food effects on intragastric pH, was previously developed and verified for esomeprazole by Liu et al. [44] in healthy subjects, accurately predicting gastric pH over 24 h.

Considering that the active compound generated in acid from esomeprazole has no chiral center and is identical to that formed from omeprazole [21], this model was utilized for omeprazole, taking into account the efficacy of omeprazole to bind H+/K+-APTase (kd) irreversibly. The dose–exposure–response relationship was assumed to be consistent across ages. We also considered the physiological changes in intragastric pH, volumes, and food effects, as well as dietary differences between adults and pediatrics. Please refer to the Supplementary Material for a detailed description of the semi-mechanistic PD model (Table S1 and Semi-mechanistic model to predict intragastric pH section). The daily average intragastric pH and duration of time above pH 4 and 6 for each age population were computed.

### 2.6. Application of the PBPK Model to Evaluate DDI on Omeprazole Exposure and Response

To investigate the potential impact of age on DDI with omeprazole as a victim drug, we simulated a hypothetical scenario where a drug fully blocks CYP3A enzymes as a perpetrator using dynamic simulations in the DDI module of GastroPlus^®^. For this simulation, we blocked CYP3A4 activity in adults and CYP3A4 and CYP3A7 activity in pediatrics. The CYP2C19 and CYP3A4 competitive inhibition and MBI processes of omeprazole were also implemented.

Initially, a single simulation was conducted to explore the impact of DDI across different age groups. Subsequently, population simulations were performed as described previously (Section 2.4). The simulation was performed for 8 days to assure the steady-state PK and PD of omeprazole. This duration allows for the evaluation of both the parent drug and its metabolites, as well as the cumulative effects on gastric acid suppression. Since the irreversible binding of PPIs like omeprazole to the H⁺/K⁺-ATPase enzyme leads to prolonged acid suppression, the predictive performance of the DDI model was evaluated by comparing the simulated plasma concentration-time profiles of omeprazole alone versus omeprazole and the perpetrator drug that fully blocks CYP3A. AUC and the maximum concentration (Cmax) ratios in the present and absence of a perpetrator were calculated using the following equations:

(1) $$DDI\;AUC\;ratio=\frac{AUC\;omeprazole\;during\;coadministration}{AUC\;omeprazole\;alone}$$

(2) $$DDI\;Cmax\;ratio=\frac{Cmax\;omeprazole\;during\;coadministration}{Cmax\;omeprazole\;alone}$$

The DDI was classified as no interaction, weak, moderate, or strong interaction based on AUC ratios. Interaction is considered weak with an AUC ratio of 1.25 to 2, moderate with an AUC ratio from 2 to 5, and strong with AUC ratios greater than 5 [45]

Simulated plasma concentration profiles obtained from the developed PBPK model, both with and without the DDI, were linked to the PD model. This link was used to explore whether age-based physiological changes in the GI tract, combined with changes in omeprazole PK, would result in an altered response. The simulated intragastric H^+^ concentration over 24 h on day 8 was utilized to evaluate the impact of DDI on the drug response using the following equation:

(3) $$DDI\;AUEC\;ratio=\frac{AUEC\;omeprazole\;during\;coadministration}{AUEC\;omeprazole\;alone}$$

Interaction is considered weak with an AUEC ratio of 1.25 to 2. An AUEC ratio between 2 and 5 indicates a moderate interaction, while AUEC ratios greater than 5 indicate a strong interaction.

### 2.7. Statistical Analysis

The distributions of the AUC values were investigated by visually inspecting the probability density histograms, quantile–quantile plots, and by using the Shapiro–Wilk statistical test. Data were classified as normal, log-normal, or nonparametric distribution. The AUC values from omeprazole and omeprazole with full CYP3A inhibition for each age group were compared using the Kruskal–Wallis Test with Dunn’s Multiple Comparison post hoc test. Significance was set at a *p* value of less than 0.05.

## 3. Results

### 3.1. PBPK Model Development and Verification of Omeprazole and Its Metabolites in Adults

The physicochemical parameters were initially obtained from literature, and the physiology of the patients was informed from the respective source of data. The key input parameters used to build the PBPK models for omeprazole, hydroxy-omeprazole, and omeprazole sulphone are summarized in Table 2, Tables S2 and S3. The model successfully predicted both the shape and magnitude of the observed profiles for omeprazole and its metabolites, as reported by Andersson et al., 1990 [27], demonstrating that the distribution and metabolism processes were well established. Predicted versus observed plasma concentrations obtained after the IV administration of 40 mg of omeprazole and oral administration of 40 and 90 mg of omeprazole are shown in Figure S1 [27].

**Figure 2:**
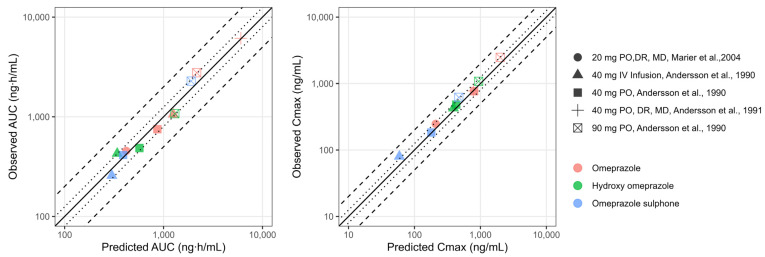
Goodness-of-fit plots of predicted versus observed pharmacokinetic metrics (AUC: panel (**A**) and Cmax; panel (**B**)) for the PBPK model in adults. The line of identity is shown as a solid line; 1.25-fold deviation is shown as a dotted line; 2-fold deviation is shown as a dashed line. Cmax: maximum concentration; AUC: area under the curve; IV: intravenous; MD: multiple dose; PO: oral administration; DR: delayed release. Andersson et al., 1990 [27], Marier et al., 2004 [28], Andersson et al., 1991 [29].

The oral PBPK model for the 40 mg formulation accurately predicted the data observed for both the parent and the metabolites. The Cmax of the parent was underpredicted with the highest dose of 90 mg. However, considering good predictions for the 40 mg capsule, the PBPK model was further verified in healthy adults after a multiple-dose regimen with plasma profiles reported by Marier et al., 2004 [28]. The inclusion of a competitive inhibition and MBI between omeprazole and its metabolites in the CYP2C19 and CYP3A4 enzymes, improved the prediction of observed plasma concentration profiles for all the subjects, as shown in Figure S2. Table 3 summarizes the parameter values used for the competitive inhibition and MBI of omeprazole, hydroxy-omeprazole, and omeprazole sulphone. The goodness-of-fit plots illustrate the alignment between the predicted versus observed pharmacokinetic metrics, specifically AUC and Cmax for the PBPK model in adults, as shown in Figure 2. Omeprazole PBPK models were verified for their contribution of CYP2C19 and CYP2C9 by simulation DDI with fluconazole, as shown in Figure S3. Our predicted DDI ratio was 5.65 aligned with the observed DDI ratio.

### 3.2. PBPK Model Extrapolation from Adults to Pediatrics

**Figure 3:**
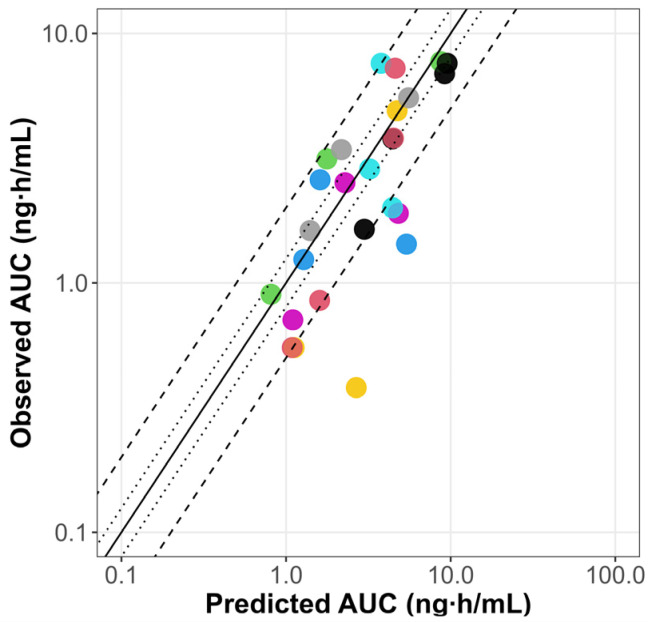
Goodness-of-fit plots of predicted versus observed omeprazole AUC for the PBPK model in pediatrics. The line of identity is shown as a solid line; 1.25-fold deviation is shown as a dotted line; 2-fold deviation is shown as a dashed line. AUC: area under the curve; iv inf: intravenous infusion. Colored dots represent predicted versus observed AUC values for different dosing regimens from clinical studies in pediatrics [31,32].

The scaled PBPK model from adults successfully predicted the pediatric PK profiles reported by Marier et al., 2004 [33], as illustrated in Figure S3. The pediatric PBPK model predicts AUC (0 to 12 h) reported by Jacqz-Aigrain et al., 1994 [30] for all the patients except for subject number 3 (Table 4). Furthermore, the PBPK model was verified using two other clinical studies [31,32] of pediatric patients after IV and oral dosing. The results are shown in Table S4. In approximately 75% of the simulations, PK parameters were within a 2-fold error of the respective observed values. The goodness-of-fit plots illustrate the alignment between the predicted versus observed AUC for the PBPK model in pediatrics, as summarized in Figure 3.

### 3.3. The Impact of Age-Dependent Physiological Changes on Omeprazole Exposure and Response

**Figure 4:**
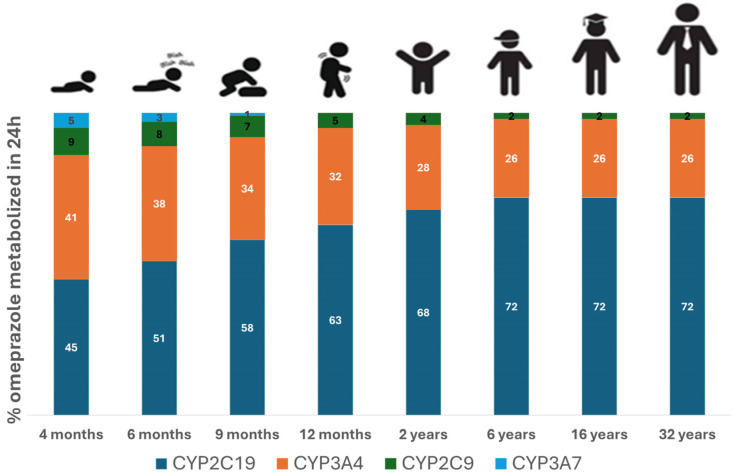
Percentage of omeprazole dose metabolized by different drug-metabolizing enzymes. Simulations were carried out using the developed PBPK model for different age groups, including 4 months, 6 months, 9 months, 1 year, 2 years, 6 years, 16 years, and 32 years of age following the administration of 10 mg omeprazole. To avoid a formulation confounding factor, all simulations were carried out using similar formulations across different age groups.

The contribution of different metabolizing enzymes to the overall metabolism of 10 mg omeprazole for various age groups is illustrated in Figure 4. In infants at 4 months old, approximately 45% of omeprazole metabolism is mediated by CYP2C19, gradually increasing to adult levels by 6 years of age. CYP2C19 becomes the major enzyme in children aged 6 years, accounting for 72% of the metabolism at that age. Conversely, metabolism via CYP3A7 decreases significantly with age and becomes negligible after the first year of life. In children under 2 years, the fraction metabolized with CYP3A4 is greater than that in older children and adults.

**Figure 5:**
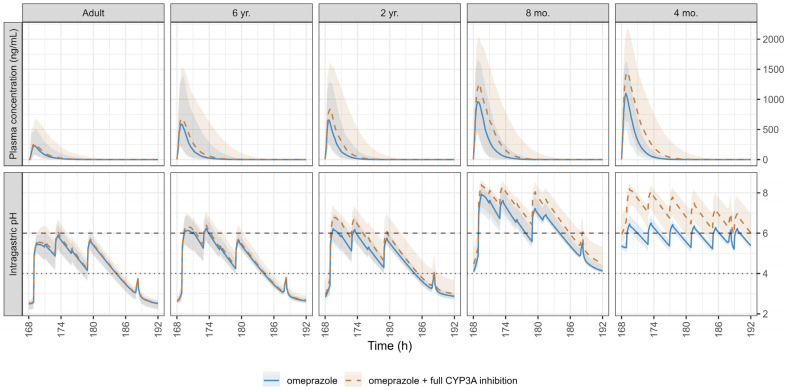
Simulated pharmacokinetic (PK) profiles with and without a hypothetical CYP3A full inhibition (**upper panel**) and the corresponding intragastric pH (**lower panel**) on day 8 following the administration of 20 mg omeprazole in adults and 0.7 mg/kg in pediatrics. The dotted line at pH 4 and the dashed line at pH 6 indicate thresholds for proton pump inhibitor treatment efficacy and safety, respectively. Data are expressed as medians (solid and dashed lines) and shaded areas represent the 5th–95th percentiles.

The results of the single simulation with and without the DDI under hypothetical full inhibition of CYP3A for five different age subjects: 37 years (representing adults) and 6 years, 2 years, 8 months, and 4 months old (representing pediatric subjects) are summarized in Table S5 and Figure S5. The simulated population PK profiles simulated with and without the CYP3A4 full inhibition, as well as the simulated intragastric pH on day 8 following the administration of 20 mg and 0.7 mg/kg of omeprazole in adults and pediatrics, respectively, are presented in Figure 5. The complete inhibition of CYP3A4 resulted in no significant changes in omeprazole exposure in adults. However, exposure nearly doubled in children aged 6 and 2 years and increased approximately 4-fold in children aged < 2 years, suggesting a moderate DDI.

**Figure 6:**
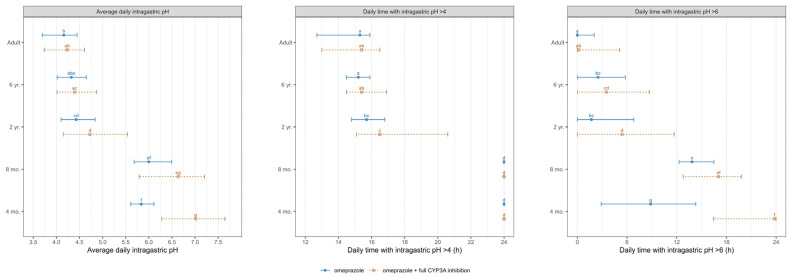
Simulated average intragastric pH and duration of pH > 4 and pH >6 over 24 h on day 8 following the administration of 20 mg omeprazole in adults and 0.7 mg/kg in pediatrics, without (blue solid line) and with (orange dashed line) a hypothetical CYP3A strong inhibition. Data are displayed as the median (●) with the 5th and 95th percentiles (—). The parameter values from omeprazole and omeprazole with full CYP3A inhibition were compared using the Kruskal–Wallis test with the Dunn’s Multiple Comparison post hoc test. Significance was set at a *p* value of less than 0.05. Groups that do not share a letter are significantly different.

The average daily intragastric pH and daily duration time of intragastric pH above 4 and 6 on day 8 following the administration of 20 mg and 0.7 mg/kg of omeprazole, with and without the DDI with a hypothetical CYP3A full inhibition, are presented in Figure 6 for both adults and pediatric populations.

**Figure 7:**
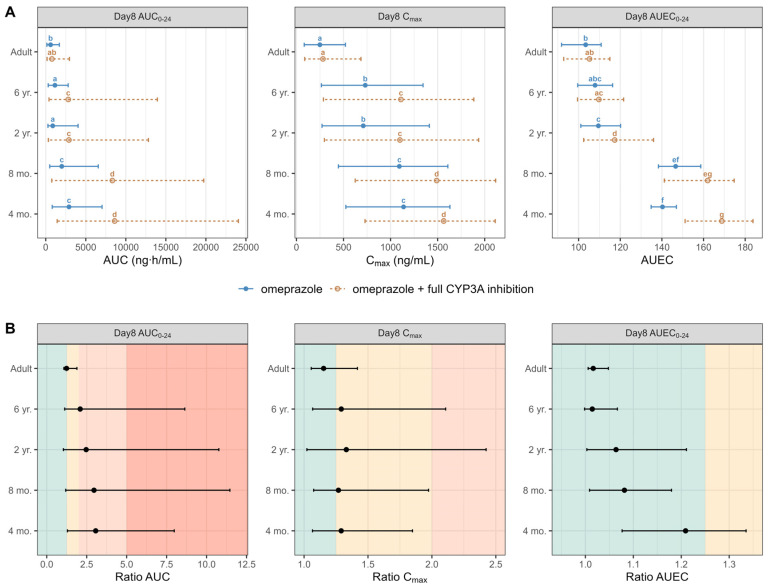
(Panel **A**) Simulated AUC, Cmax, and AUEC on day 8 following the administration of 20 mg omeprazole in adults and 0.7 mg/kg omeprazole pediatrics without (solid blue line) and with (dashed orange line) the DDI under a hypothetical full CYP3A inhibition. Data are displayed as the median (●) and the 5th and 95th percentiles (—). The parameter values from omeprazole and omeprazole with the full CYP3A inhibition of each group are compared by the Kruskal–Wallis test with the Dunn’s Multiple Comparison post hoc test. Significance was set at a *p* value of less than 0.05. (Panel **B**) Calculated DDI AUC, Cmax, and AUEC ratios on day 8 following the administration of 20 mg and 0.7 mg/kg of omeprazole, in adults and pediatrics, respectively, without and with the DDI under a hypothetical full CYP3A inhibition. Data are displayed as the median (●) and the 5th and 95th percentiles (—). The green area indicates no DDI interaction (ratio < 1.25), the yellow area indicates a weak DDI interaction (ratio range from 1.25 to 2), the orange area indicates a moderate DDI interaction (ratio range 2 to 5), while the dark orange area indicates a strong DDI interaction (ratio > 5). Groups that do not share a letter are significantly different.

Figure 7 and Table S6 summarize the DDI dynamic population simulation (100 subjects) of PK and PD parameters and ratios for omeprazole administrated alone and as a victim with a hypothetical drug that fully blocks the CYP3A enzymes as a perpetrator in healthy different ages populations. In the absence of CYP3A inhibition, significantly higher omeprazole exposure and the subsequent response at therapeutic doses were observed in children aged ≤ 2 years compared to older children and adults, indicating potential dose adjustment requirements for this age group. A non-significant increase in omeprazole exposure was noted in adults after administering omeprazole with full CYP3A inhibition. However, the drug-driven phenoconversion in pediatrics due to the blockade of the CYP3A led to an almost 2-fold increase in children aged ≤ 6 and >2 years and an almost 4-fold increase in children aged ≤ 2 years, highlighting the need for specific DDI recommendations in younger pediatrics. Overall, our predictions indicated a significantly elevated intragastric pH in infants under 1 year of age compared to adults following the administration of omeprazole. Simulations also indicated that the daily duration time with pH > 4 and pH > 6 was significantly higher in young infants under 1 year of age than in adults, exposing those populations to a higher risk of PPI adverse events.

## 4. Discussion

In this study, we successfully developed and validated a whole-body PBPK model for omeprazole and its metabolites hydroxy omeprazole and omeprazole sulfone across adult and pediatric populations. This model provides valuable insights into the exposure–response relationship following omeprazole administration in different age groups. We identified significant age-dependent differences in the contribution of various CYP enzymes, including CYP2C19, CYP3A4, CYP2C9, and CYP3A7, to omeprazole metabolism. In children under 1 years of age, CYP3A4 as well as CYP2C19 were the major enzymes involved in metabolism, whereas CYP2C19 became the dominant enzyme by the age of 6, accounting for 72% of the total metabolism. These age-dependent shifts in enzyme activity are crucial for understanding the PK of omeprazole and may inform age-specific dosing recommendations, including those for DDI scenarios.

One of the significant findings from this study is the markedly elevated intragastric pH levels observed in infants and children under 1 year of age after omeprazole administration. This result underscores the need for age-appropriate dosing strategies, as prolonged intragastric pH elevation could increase the risk of adverse effects, such as bacterial overgrowth and calcium malabsorption. The elevated pH observed in young children is consistent with clinical studies showing that PPIs, such as omeprazole, can significantly alter gastric acidity in this population [19], potentially leading to unintended consequences if dosing is not carefully tailored.

Compared to traditional population PK models, PBPK modeling offers significant advantages by explicitly accounting for developmental changes in enzyme expression and activity, and allowing for the mechanistic evaluation of DDIs [28,51]. Even though leveraging other PBPK models for omeprazole mainly focused on biopharmaceutics [52,53] to inform the model parameters, our model incorporated auto-inhibition mechanisms, including competitive inhibition and mechanism-based inactivation (MBI) between omeprazole and its metabolites in the CYP2C19 and CYP3A4 enzymes [14]. By integrating these interactions, the PBPK model accurately captured the nonlinear PK of omeprazole. This highlights the importance of considering metabolic interactions when predicting drug exposure, especially in pediatric populations with differing enzyme ontogeny compared to adults, making virtual testing DDI possible in populations where clinical DDI studies are unrealistic We also acknowledge that the impact of genetic variability and disease states (e.g., Child–Pugh score) on omeprazole PK and PD would be an interesting addition. However, it is important to note that these factors were outside the scope of the current study, which specifically focused on the impact of DDIs in pediatric populations. Future work could explore the integration of genetic variability and disease states into the model for a more comprehensive understanding of omeprazole PK/PD in different patient populations.

In this study, we modeled the different formulations used in the reference studies from the literature, which included formulations such as such as enteric-coated capsules and granules. Given that GastroPlus does not have the enteric coated capsules as a formulation, we used enteric coated tablets instead. However, for the purpose of model application and simplification during simulations, we decided to use the suspension formulation for pediatric populations and tablet formulation for adults. We acknowledge that omeprazole is often available as an enteric-coated formulation, which delays drug release until it reaches the small intestine, thus influencing the absorption profile and PK of the drug. The delayed absorption associated with enteric coatings can affect the time to peak concentration and the overall drug exposure. While we did consider enteric-coated formulations in the initial simulations for the different studies, this factor was not fully incorporated in all simulations for the DDI evaluation. Given that the model is able to reproduce the difference formulations as demonstrated during the verification step, it can be applied to specifically evaluate DDI scenarios with the enteric-coated formulations.

The adult PBPK model provides a consistent representation of omeprazole, hydroxy omeprazole, and omeprazole sulphone kinetic disposition following IV and the extravascular administration of single and multiple doses of omeprazole from various studies with predicted-to-observed AUC and Cmax ratios within the 1.25-fold. CYP3A7 activity in the pediatric model improved its performance, particularly for younger children. CYP3A7 plays a critical role during fetal development and decreases sharply after birth, which explains the observed improvement in the model’s performance when its activity was accounted for in infants. CYP3A7 activity is high during embryogenesis and fetal life, decreasing rapidly during the first week of life to only 10% of newborn levels between 3 and 12 months of age [54]. This explains the improved performance of our PBPK model after accounting for CYP3A7 in younger children.

Additionally, the contributions of CYP3A4 to omeprazole metabolism is significantly higher in children aged 1 year and younger compared to children aged 6 years and adults, highlighting the impact of age-dependent metabolic pathways on omeprazole metabolism. Our findings align with previous PK studies [55,56] that suggested that the activity of CYP3A4 isoform is markedly increased in young infants and children compared to adolescents and adults. Overall, in approximately 75% of the pediatric PBPK simulations, predicted PK parameters were within a 2-fold error of the observed values, indicating good predictive model performance.

This study confirmed that the CYP2C19 enzyme is the principal enzyme involved in omeprazole metabolism in adults and older children [55,57,58], with CYP3A4 contributing to a lesser degree. This highlights the importance of considering metabolic interactions when predicting drug exposure, especially in pediatric populations with differing enzyme expression compared to adults. Given that CYP2C19 is highly polymorphic, genotype-guided doses could be beneficial for 1-year-old children and upwards to adults, where CYP2C19 plays a significant role in omeprazole metabolism. However, in younger children, particularly those less than 1 year old, the contribution of CYP2C19 to omeprazole metabolism may not be sufficient to warrant genotype-guided dosing, as other enzymes like CYP3A4 dominate in this age group. This underscores the importance of considering both enzyme expression and activity during pediatric drug development.

Although the PBPK model well captures the age-dependent differences in omeprazole metabolism, it is important to acknowledge the limitations of this approach. The lack of detailed in vivo data on enzyme kinetics in infants and children required us to make assumptions, such as using the relative Km and Vmax values for CYP3A7 calculated from those for CYP3A4. Despite this assumption, our model predictions closely matched observed plasma concentration–time profiles, suggesting that PBPK modeling can support decisions on pediatric drug dosing. Furthermore, while the model accounted for the ontogeny of enzymes like CYP3A7 and CYP3A4, more detailed data on the maturation of these enzymes over time is needed to refine predictions for different pediatric subgroups further.

In addition to the PK considerations, this study evaluated the impact of age on omeprazole’s PD, specifically its effect on intragastric pH. By the age of 4 months, the gastric proton pump (H^+^/K^+^-ATPase) responsible for acid secretion in the stomach is considered to be functionally mature [59]. This means that omeprazole can effectively inhibit acid production from this age onward, similar to its effect in older children and adults. Therefore, a similar dose–exposure–response relationship was assumed across ages. The PD activity was attributed to the parent compound, omeprazole, as its metabolites do not affect gastric acid secretion [57]. We used an indirect response model incorporating the synthesis and degradation of H^+^/K^+^-ATPase, food effects on gastric acid concentration, and circadian rhythm [44]. This model was previously applied to predict intragastric pH after omeprazole administration in other special populations [60]. In healthy volunteers, the reported mean intragastric pH over 24 h and the mean percent durations of time with intragastric pH > 4 after a single dose of 20 mg omeprazole were 1.8 and 30.4%, respectively. Following multiple 20 mg doses, these values increased to 3.5 ± 1.0 and 48.7 ± 20.5%, respectively [61]. These records were in agreement with our simulations (refer to Figure 6).

In infants and children, an initial oral dosing of 0.7 to 3.3 mg/kg of omeprazole is considered effective and well tolerated for esophagitis and GERD [6,43]. Our PD simulations in children receiving a dose of 0.7 mg/kg led to intragastric pH above 4 in infants less than 1 year of age 100% of the daily time, which is significantly higher than adults (Figure 5 and Figure 6). Our simulations are based on real-life scenarios, considering infants fed with mother’s milk or formula regularly. Infants are typically fed more frequently than older children, leading to a more constant buffering effect on stomach acidity by milk or formula. Additionally, the high fluid intake relative to body size in infants can dilute gastric contents, resulting in a higher pH. The study conducted by Jonathan Bishop [62] in children younger than 2 years (median 7.75 months, range 1.25–20 months) treated with a median omeprazole dose of 1.05 mg/kg/day showed more than 97% of the 24 h period with gastric pH more than 4, in agreement with our findings. However, in another short-term study [31], a daily dose of 40 mg/1.73 m^2^ (1.17 mg/kg), higher than the equivalent recommended oral dose in children, administered in fasting 4.5 to 27 months patients, was required to achieve a gastric pH more than 4 during more than 90% of the 24 h period after omeprazole IV administration. This may be attributed to the fasting state, as the gastric pH is susceptible to feeding schedules; the postprandial pH in an infant’s stomach stays above 4.5 for about two hours, while the gastric pH under the fasted state is strongly acidic [63].

The efficacy and safety of omeprazole in adults have been demonstrated over the last 30 years [64,65]. However, its safe administration in children has not yet been fully resolved, although it has been investigated at the clinical level, and its therapeutic effectiveness for the pediatric population has been validated [66]. PPIs are associated with safety concerns where elevation of intragastric pH above 4 can cause hypochlorhydria and bacterial overgrowth in the stomach, increasing the risk of gastrointestinal infections and bone fractures due to calcium malabsorption [19]. Our simulations revealed a long daily duration for the intragastric pH being above 6 in 9-month-old and 4-month-old infants compared to adults and older children. These observations emphasize the recommendation against using PPIs without a clear indication in young children.

This study integrated the mechanisms of drug metabolism and enzyme ontogeny to emphasize the importance of the age-based evaluation of DDIs. The interaction of omeprazole with strong CYP3A4 inhibitors, such as itraconazole, did not significantly increase omeprazole exposure in adults. However, drug-driven phenoconversion in pediatrics due to the CYP3A blockade led to an almost 4-fold increase in children under 2 years, highlighting the need for specific DDI recommendations in younger pediatrics. Infants receiving omeprazole with strong CYP3A4 inhibitors may thus require dose adjustments. Although this increase in exposure did not result in significant changes in effect, it is important to highlight that the intragastric pH in infants and young children was already above 4 throughout the full dosing interval. The impact of the interaction at lower doses; however, may lead to different conclusions, potentially altering the clinical outcomes and necessitating dose adjustments in this population. Given the substantial differences in the expression and activity of metabolic enzymes across age groups, particularly in pediatrics, it is critical to specifically evaluate DDIs in this population to ensure accurate and safe dose recommendations. Since conducting DDI studies in pediatric populations presents significant ethical and practical challenges, leveraging PBPK models offers a promising solution. These models enable the prediction of drug behavior and potential interactions in pediatric patients, filling the gap where direct clinical studies are not feasible. By incorporating age-dependent changes in metabolic pathways and enzyme activity, PBPK models provide a robust framework for assessing DDIs in children, thereby helping to tailor dose recommendations and minimize risks in this vulnerable population.

## 5. Conclusions

The PBPK model was able to predict the shape and magnitude of the observed profiles for both omeprazole and its metabolites in adults and pediatrics. The final PBPK model included the competitive inhibition and MBI of CYP2C19 and CYP3A4 by omeprazole and its metabolites. By accounting for age-dependent changes in metabolic pathways, the PBPK model enabled pharmacokinetics (PK), a priori predictions across different age populations. Linking PK to a response model that considers age-based physiological changes allows for a quantitative description of alterations in intragastric pH across age populations. By virtually assessing dosing scenarios, particularly in populations where clinical studies pose challenges, the PBPK-PD model offers a feasible alternative to empirical dosage decisions and provides guidance for the recommended dose of omeprazole. Given the age-dependent variability in metabolic enzyme expression and activity, evaluating DDIs in pediatrics is essential.

## Figures and Tables

**Table 1:** Clinical PK data used for omeprazole and the metabolite PBPK model development and verification.

	Study (Reference)	Number Sex	Dosage Regimen	Route	Age [Years] Mean ± SD (Range)	Body Weight [kg] Mean ± SD (Range)
Healthy adult PBPK model development	[27]	10 M	40 mg single dose 40 mg and 90 mg single dose	IV PO	(19–27)	(70–86)
Healthy adult PBPK model verification	[28]	18M	20 mg delayed-release capsules multiple doses	PO	36.8 ± 5.6 (26–45)	71.0 ± 7.6 (58.5–84.4)
	[29]	12M	40 mg delayed-release granules multiple doses	PO	(23–29)	(67–86)
Pediatric extrapolation of the PBPK model	[28]	6M/6F	10 to 20 mg delayed-release capsules multiple doses	PO	6.1 ± 4.4 (0.5–13)	24.2 ± 16.5 (6.0–64.2)
Pediatric PBPK model verification	[30]	5 *	60 to 80 mg/1.73 m^2^ loading dose followed by 40 mg * 1.73 m^2^ at 12 h intervals,	Slow bolus or over 15 min IV infusion		
	[31]	6M/3F	20 to 40 mg/1.73 m^2^ once daily	IV infusion (1 h)	(0.4–2.3)	
	[32]	25	the “healing dose” of omeprazole in each child was that which controlled pathological acid reflux	PO	(1–16)	(10.7–68)

M: male; F: female; IV: intravenous; PO: per oral. * Only the children without concomitant medication were chosen.

**Table 2:** Key physicochemical and biopharmaceutical parameters for omeprazole PBPK model.

Parameter	Value	Reference
Molecular weight	345.4 g/mol	[42,46]
logP	2.23	[46]
Diffusion coefficient	0.71 × 10^−5^ cm^2^/s	ADMET Predictor v.9.5
pKa	14.7 (Acid) 7.1 (Base)	[47]
Reference solubility	0.0823 mg/mL at pH = 7.4	[48]
Solubility Factor	125.54	ADMET Predictor v.9.5
FaSSIF solubility	0.27 mg/mL at pH = 6.5	[49]
FeSSIF solubility	0.22 mg/mL at pH = 5.0	[49]
Human effective permeability (P_eff_)	12 × 10^−4^ cm/s	Fitted
Particle radius	25 μm	GastroPlus v.9.8 default
Precipitate radius	1 μm	GastroPlus v.9.8 default
Drug particle density	1.2 g/mL	GastroPlus v.9.8 default
Mean precipitation time	900 s	GastroPlus v.9.8 default
Blood–plasma concentration ratio (R_bp_)	0.6	[50]
Fraction unbound in plasma (Fup)%	4.9852%	[50]
Formation of 5-O-desmethyl metabolite		
CYP2C19 K_m,u_ (PBPK)	0.811 mg/L	[34]
CYP2C19 V_max_ (PBPK)	5.115 × 10^−4^ mg/s/mg enzyme	Initially informed from in vitro [34] then fitted
CYP2C9 K_m,u_ (PBPK)	73.92 mg/L	[34]
CYP2C9 V_max_ (PBPK)	7.97 × 10^−5^ mg/s/mg enzyme	[34]
CYP3A4 K_m,u_ (PBPK)	181 mg/L	[34]
CYP3A4 V_max_ (PBPK)	3.637 × 10^−3^ mg/s/mg enzyme	Initially informed from in vitro [34] then fitted
CYP3A7 K_m,u_ (PBPK)	923.1 mg/L	Calculated from K_m_ for CYP3A4 [41]
CYP3A7 V_max_ (PBPK)	9.09 × 10^−4^ mg/s/mg enzyme	Calculated from V_max_ for CYP3A4 [41]
Formation of Hydroxy metabolite		
CYP2C19 K_m,u_ (PBPK)	1.657 mg/L	[32]
CYP2C19 V_max_ (PBPK)	3.67 × 10^−3^ mg/s/mg enzyme	Initially informed from in vitro [32] then fitted
CYP2C9 K_m,u_ (PBPK)	141.3 mg/L	[32]
CYP2C9 V_max_ (PBPK)	1.803 × 10^−3^ mg/s/mg enzyme	Initially informed from in vitro [32] then fitted
CYP3A4 K_m,u_ (PBPK)	117.4 mg/L	[32]
CYP3A4 V_max_ (PBPK)	8.39 × 10^−4^ mg/s/mg enzyme	[32]
CYP3A7 K_m,u_ (PBPK)	598.23 mg/L	Calculated from K_m_ for CYP3A4 [41]
CYP3A7 V_max_ (PBPK)	2.10 × 10^−4^ mg/s/mg enzyme	Calculated from V_max_ for CYP3A4 [41]
Formation of Sulphone metabolite		
CYP3A4 K_m,u_ (PBPK)	28.57 mg/L	[34]
CYP3A4 V_max_ (PBPK)	1.5 × 10^−3^ mg/s/mg enzyme	Initially informed from in vitro [34] then fitted
CYP3A4 K_m,u_ (Gut)	28.57 mg/L	[34]
CYP3A4 V_max_ (Gut)	0.921 mg/s/mg enzyme	Initially informed from in vitro [34] then fitted
CYP3A7 K_m,u_ (PBPK)	145.7 mg/L	Calculated from K_m_ for CYP3A4 [41]
CYP3A7 V_max_ (PBPK)	3.75 × 10^−4^ mg/s/mg enzyme	Calculated from V_max_ for CYP3A4 [41]

FaSSIF: fasted state simulated intestinal fluid; FeSSIF: fed state simulated intestinal fluid.

**Table 3:** In vitro inhibitory parameters of omeprazole and its metabolites for CYP2C19 and CYP3A4.

CYP450 target	Inhibitor	Parameters			References
		IC_50_	K_I_	K_inact_	
CYP2C19	Omeprazole	8.4 µM	1.1 µM	0.048 min^−1^	[14,15]
	Hydroxy omeprazole	39 µM			[14]
	Omeprazole sulphone	5.1 µM	5.7 µM	0.015 min^−1^	[14]
CYP3A4	Omeprazole	40 µM	52 µM	0.029 min^−1^	[14]
	Hydroxy omeprazole	21 µM			[14]
	Omeprazole sulphone	8 µM			[14]

**Table 4:** Predicted vs. observed (Jacqz-Aigrain et al., 1994 [30]) AUC_0–12_ across different pediatrics patients.

Subject	Age (Years)	Dose (mg)	Body Weight (kg) ^a^	Observed AUC_0–12_ (µg∙h/mL)	Predicted AUC_0–12_ (µg∙h/mL) Without CYP3A7, *n* = 1 Subject	Predicted AUC_0–12_ (µg∙h/mL) with CYP3A7, *n* = 1 Subject	Predicted AUC_0–12_ (µg∙h/mL) with CYP3A7 (Min–Max), *n* = 25 Subjects
1	0.3	6	4.8	6.42	16	10.55	(1.14–11.11)
2	0.7	8	7.0	2.56	4.4	3.05	(0.41–7.53)
3	0.8	9	9.7	1.48	3.13	2.36	(0.57–12.1)
4	1.6	9	7.0	5.24	5.1	5.03	(0.84–16.2)

Subject 6 was removed from analysis due to administration of erroneous dosage. ^a^ Calculated using the Du Bois equation [42].

## Data Availability

Data are contained within the article and Supplementary Materials.

## Supplementary Materials

The following supporting information can be downloaded at: https://www.mdpi.com/article/10.3390/pharmaceutics17030373/s1, References [67,68,69,70,71,72,73,74,75,76] are cited in the supplementary materials. Table S1. Summary of pharmacodynamic model parameters, based on Liu et al. [44]. Table S2. Key physicochemical and biopharmaceutical parameters for metabolite Hydroxy-Omeprazole. Table S3. Key physicochemical and biopharmaceutical parameters for metabolite Omeprazole Sulphone. Table S4. Initial physiologically based pharmacokinetic (PBPK) Model - Simulated versus observed PK parameters for omeprazole intravenous and oral doses in children. Table S5. DDI dynamic single simulation- Simulated PK and PD parameters and ratios for omeprazole administrated alone and as a victim with a hypothetical drug which fully blocks the CYP 3A enzymes as a perpetrator in healthy subjects. Table S6. DDI dynamic population simulation (100 subjects)- Simulated PK and PD parameters and ratios for omeprazole administrated alone and as a victim with a hypothetical drug which fully blocks the CYP 3A enzymes as a perpetrator in healthy different ages populations. Figure S1. PBPK model development; Predicted versus observed plasma concentrations obtained after intravenous administration of 40 mg of omeprazole (a), oral administration of 40 mg of omeprazole (b) and oral administration of 90 mg of omeprazole (c). Omeprazole (grey), hydroxy-omeprazole (blue) and omeprazole sulphone (orange). Observed data; Andersson et al, 1990 [27]. Figure S2. Verification of omeprazole PBPK model in adults after multiple dosing administration. Figure S3. Predicted versus observed plasma concentration profiles obtained after oral administration of 16.7 mg of omeprazole in 6-year-old children with inclusion of autoinhibition and mechanism-based inactivation processes. Observed data; Marier et al., 2004 [28]. Figure S4. Verification of the contribution of CYP2C19 for omeprazole metabolism in omeprazole PBPK model in adults. Figure S5. Single simulation results of different age subjects after the administration of omeprazole alone and in combination with CYP3A inhibition. Section: Semi-mechanistic model to predict intragastric pH.

## References

[1] Lima J.J., Thomas C.D., Barbarino J., Desta Z., Van Driest S.L., El Rouby N., Johnson J.A., Cavallari L.H., Shakhnovich V., Thacker D.L. (2021). Clinical Pharmacogenetics Implementation Consortium (CPIC) Guideline for CYP2C19 and Proton Pump Inhibitor Dosing. Clin. Pharmacol. Ther..

[2] Boulton K.H.A., Dettmar P.W. (2022). A Narrative Review of the Prevalence of Gastroesophageal Reflux Disease (GERD). Ann. Esophagus.

[3] Nirwan J.S., Hasan S.S., Babar Z.-U.-D., Conway B.R., Ghori M.U. (2020). Global Prevalence and Risk Factors of Gastro-Oesophageal Reflux Disease (GORD): Systematic Review with Meta-Analysis. Sci. Rep..

[4] Cicala M. (2013). Proton Pump Inhibitor Resistance, the Real Challenge in Gastro-Esophageal Reflux Disease. WJG.

[5] El-Serag H., Becher A., Jones R. (2010). Systematic Review: Persistent Reflux Symptoms on Proton Pump Inhibitor Therapy in Primary Care and Community Studies. Aliment. Pharmacol. Ther..

[6] Klinkenberg-knol E.C., Nelis F., Dent J., Snel P., Mitchell B., Prichard P., Lloyd D., Havu N., Frame M.H., Roma J. (2000). Long-Term Omeprazole Treatment in Resistant Gastroesophageal Reflux Disease: Efficacy, Safety, and Influence on Gastric Mucosa. Gastroenterology.

[7] Andersson T. (1996). Pharmacokinetics, Metabolism and Interactions of Acid Pump Inhibitors. Clin. Pharmacokinet..

[8] Lind T., Cederberg C., Ekenved G., Haglund U., Olbe L. (1983). Effect of Omeprazole--a Gastric Proton Pump Inhibitor--on Pentagastrin Stimulated Acid Secretion in Man. Gut.

[9] Cederberg C., Andersson T., Skånberg I. (1989). Omeprazole: Pharmacokinetics and Metabolism in Man. Scand. J. Gastroenterol..

[10] El-Kimary E.I., Ragab M.A.A. (2022). Recent Analytical Methodologies for the Determination of Omeprazole and/or Its Active Isomer Esomeprazole in Different Matrices: A Critical Review. Crit. Rev. Anal. Chem..

[11] Shin J.M., Kim N. (2013). Pharmacokinetics and Pharmacodynamics of the Proton Pump Inhibitors. J. Neurogastroenterol. Motil..

[12] Yu L.-Y., Sun L.-N., Zhang X.-H., Li Y.-Q., Yu L., Yuan Z.-Q.-Y., Meng L., Zhang H.-W., Wang Y.-Q. (2017). A Review of the Novel Application and Potential Adverse Effects of Proton Pump Inhibitors. Adv. Ther..

[13] Angiolillo D.J., Gibson C.M., Cheng S., Ollier C., Nicolas O., Bergougnan L., Perrin L., Lacreta F.P., Hurbin F., Dubar M. (2011). Differential Effects of Omeprazole and Pantoprazole on the Pharmacodynamics and Pharmacokinetics of Clopidogrel in Healthy Subjects: Randomized, Placebo-Controlled, Crossover Comparison Studies. Clin. Pharmacol. Ther..

[14] Shirasaka Y., Sager J.E., Lutz J.D., Davis C., Isoherranen N. (2013). Inhibition of CYP2C19 and CYP3A4 by Omeprazole Metabolites and Their Contribution to Drug-Drug Interactionss. Drug Metab. Dispos..

[15] Zvyaga T., Chang S.Y., Chen C., Yang Z., Vuppugalla R., Hurley J., Thorndike D., Wagner A., Chimalakonda A., Rodrigues A.D. (2012). Evaluation of Six Proton Pump Inhibitors as Inhibitors of Various Human Cytochromes P450: Focus on Cytochrome P450 2C19. Drug Metab. Dispos..

[16] Ko J.-W., Sukhova N., Thacker D., Chen P., Flockhart D.A. (1997). Evaluation of Omeprazole and Lansoprazole as Inhibitors of Cytochrome P450 Isoforms. Drug Metab. Dispos..

[17] U.S. Food and Drug Administration (2023). Drug Development and Drug Interactions: Table of Substrates, Inhibitors, and Inducers.

[18] Losurdo G., Caccavo N.L.B., Indellicati G., Celiberto F., Ierardi E., Barone M., Di Leo A. (2023). Effect of Long-Term Proton Pump Inhibitor Use on Blood Vitamins and Minerals: A Primary Care Setting Study. J. Clin. Med..

[19] Lassalle M., Zureik M., Dray-Spira R. (2023). Proton Pump Inhibitor Use and Risk of Serious Infections in Young Children. JAMA Pediatr..

[20] Wagner C., Zhao P., Pan Y., Hsu V., Grillo J., Huang S., Sinha V. (2015). Application of Physiologically Based Pharmacokinetic (PBPK) Modeling to Support Dose Selection: Report of an FDA Public Workshop on PBPK. CPT Pharmacom Syst. Pharma.

[21] Shin J.M., Sachs G. (2009). Long-Lasting Inhibitors of the Gastric H,K-ATPase. Expert Rev. Clin. Pharmacol..

[22] Shebley M., Sandhu P., Emami Riedmaier A., Jamei M., Narayanan R., Patel A., Peters S.A., Reddy V.P., Zheng M., De Zwart L. (2018). Physiologically Based Pharmacokinetic Model Qualification and Reporting Procedures for Regulatory Submissions: A Consortium Perspective. Clin. Pharma Ther..

[23] GastroPlus® PBPK & PBBM Modeling and Simulation. https://www.simulations-plus.com/software/gastroplus/.

[24] Rackauckas C., Ma Y., Noack A., Dixit V., Mogensen P.K., Elrod C., Tarek M., Byrne S., Maddhashiya S., Calderón J.B.S. (2020). Accelerated Predictive Healthcare Analytics with Pumas, A High Performance Pharmaceutical Modeling and Simulation Platform. BioRxiv.

[25] R Core Team (2023). R: A Language and Environment for Statistical Computing. R Foundation for Statistical Computing.

[26] R Studio Team (2020). RStudio: Integrated Development for R.

[27] Andersson T., Cederberg C., Reggtrdh C.-G., Skgtnberg I. (1990). European Doumal of (Pharmacokinetics of Various Single Intravenous and Oral Doses of Omeprazole. Eur. J. Clin. Pharmacol..

[28] Marier J.-F., Dubuc M.-C., Drouin E., Alvarez F., Ducharme M.P., Brazier J.-L. (2004). Pharmacokinetics of Omeprazole in Healthy Adults and in Children with Gastroesophageal Reflux Disease. Ther. Drug Monit..

[29] Andersson T., Cederberg C., Heggelund A., Lundborg P. (1991). The Pharmacokinetics of Single and Repeated Once-Daily Doses of 10, 20 and 40mg Omeprazole as Enteric-Coated Granules. Drug Investig..

[30] Jacqz-Aigrain E., Andre J., Bellaich M., Faure C., Navarro J., Rohrlich P., Baudouin V. (1994). Pharmacokinetics of Intravenous Omeprazole in Children. Eur. J. Clin. Pharmacol..

[31] Faure C., Michaud L., Shaghaghi E.K., Popon M., Turck D., Navarro J., Jacqz-Aigrain E. (2001). Intravenous Omeprazole in Children: Pharmacokinetics and Effect on 24-Hour Intragastric pH. J. Pediatr. Gastroenterol. Nutr..

[32] Andersson T., Hassall E., Ch M.B.B., Shepherd R., Radke M., Marcon M., Dalväg A., Martin S., Behrens R., Koletzko S. (2000). Pharmacokinetics of Orally Administered Omeprazole in Children. Am. J. Gastroenterol..

[33] Lukacova V., Parrott N., Lave T., Fraczkiewicz G., Bolger M. General Approach to Calculation of Tissue:Plasma Partition Coefficients for Physiologically Based Pharmacokinetic (PBPK) Modeling. Proceedings of the Simulations Plus.

[34] Abelo A.A., Andersson T.B., Bredberg U., Skånberg I., Weidolf L. (1999). Stereoselective Metabolism by Human Liver Cyp Enzymes of a Substituted Benzimidazole. Drug Metab. Dispos..

[35] Andersson T., Hassan-Alin M., Hasselgren G., Röhss K., Weidolf L. (2001). Pharmacokinetic Studies with Esomeprazole, the (S)-Isomer of Omeprazole. Clin. Pharmacokinet..

[36] Abelo A.A., Andersson T.B., Antonsson M., Naudot A.K., Skånberg I., Weidolf L. (1994). Stereoselective Metabolism of Omeprazole by Human Cytochrome P450 Enzymes. Drug Metab. Dispos..

[37] Andersson T., Miners J., Veronese M., Birkett D. (1994). Identification of Human Liver Cytochrome P450 Isoforms Mediating Secondary Omeprazole Metabolism. Br. J. Clin. Pharma.

[38] Kang B.C., Yang C.Q., Cho H.K., Suh O.K., Shin W.G. (2002). Influence of Fluconazole on the Pharmacokinetics of Omeprazole in Healthy Volunteers. Biopharm. Drug Disp..

[39] Williams J.A., Ring B.J., Cantrell V.E., Jones D.R., Eckstein J., Ruterbories K., Hamman M.A., Hall S.D., Wrighton S.A. (2002). Comparative Metabolic Capabilities of CYP3A4, CYP3A5, and CYP3A7. Drug Metab. Dispos..

[40] Lacroix D., Sonnier M., Moncion A., Cheron G., Cresteil T. (1997). Expression of CYP3A in the Human Liver — Evidence That the Shift between CYP3A7 and CYP3A4 Occurs Immediately After Birth. Eur. J. Biochem..

[41] Kovar L., Schräpel C., Selzer D., Kohl Y., Bals R., Schwab M., Lehr T. (2020). Physiologically-Based Pharmacokinetic (PBPK) Modeling of Buprenorphine in Adults, Children and Preterm Neonates. Pharmaceutics.

[42] Du Bois D., Du Bois E.F. (1989). A Formula to Estimate the Approximate Surface Area If Height and Weight Be Known. 1916. Nutrition.

[43] Pacifici G.M. (2022). Clinical Pharmacology of Omeprazole in Infants and Children. J. Drug Des. Res..

[44] Liu D., Yang H., Jiang J., Nagy P., Shen K., Qian J., Hu P. (2016). Pharmacokinetic and Pharmacodynamic Modeling Analysis of Intravenous Esomeprazole in Healthy Volunteers: Esomeprazole in Healthy Volunteers. J. Clin. Pharmacol..

[45] U.S. Department of Health and Human Services, Food and Drug Administration, Center for Drug Evaluation and Research (CDER), Center for Biologics Evaluation and Research (CBER) (2024). M12 Drug Interaction Studies: Guidance for Industry.

[46] National Center for Biotechnology Information (2004). PubChem Compound Summary for CID 4594, Omeprazole.

[47] Yang R., Schulman S.G., Zavala P.J. (2003). Acid–Base Chemistry of Omeprazole in Aqueous Solutions. Anal. Chim. Acta.

[48] Singh R., Saraf S. (2003). Spectrophotometric Estimation of Omeprazole in Pharmaceutical Dosage Form. Res. J. Pharm. and Tech..

[49] Handin N. (2021). Proteomics Informed Investigation of Human Hepatocytes and Liver Tissue. Digital Comprehensive Summaries of Uppsala Dissertations from the Faculty of Pharmacy.

[50] Regårdh C.-G., Gabrielsson M., Hoffman K.-J., Löfberg I., Skånberg I. (1985). Pharmacokinetics and Metabolism of Omeprazole in Animals and Man—An Overview. Scand. J. Gastroenterol..

[51] Regårdh C.G., Andersson T., Lagerström P.O., Lundborg P., Skånberg I. (1990). The Pharmacokinetics of Omeprazole in Humans—A Study of Single Intravenous and Oral Doses. Ther. Drug Monit..

[52] Yang R., Lin Y., Chen K., Huang J., Yang S., Yao A., Yang X., Lei D., Xiao J., Yang G. (2024). Establishing Virtual Bioequivalence and Clinically Relevant Specifications for Omeprazole Enteric-Coated Capsules by Incorporating Dissolution Data in PBPK Modeling. AAPS J..

[53] Segregur D., Mann J., Moir A., Karlsson E.M., Dressman J. (2021). Prediction of Plasma Profiles of a Weakly Basic Drug after Administration of Omeprazole Using PBPK Modeling. Eur. J. Pharm. Sci..

[54] De Wildt S.N., Kearns G.L., Leeder J.S., Van Den Anker J.N. (1999). Cytochrome P450 3A Ontogeny and Drug Disposition. Clin. Pharmacokinet. (Drug Dispos.).

[55] Kearns G.L., Andersson T., James L.P., Gaedigk A., Kraynak R.A., Abdel-Rahman S.M., Ramabadran K., Van Den Anker J.N. (2003). Pediatric Pharmacology Research Unit Network Omeprazole Disposition in Children Following Single-Dose Administration. J. Clin. Pharma.

[56] Leeder J.S., Kearns G.L. (1997). Pharmacogenetics in Pediatrics. Pediatr. Clin. North. Am..

[57] Dean L., Kane M., Pratt V.M., Scott S.A., Pirmohamed M., Esquivel B., Kattman B.L., Malheiro J.A. (2012). Omeprazole Therapy and CYP2C19 Genotype.

[58] Shirai N., Furuta T., Moriyama Y., Okochi H., Kobayashi K., Takashima M., Xiao F., Kosuge K., Nakagawa K., Hanai H. (2001). Effects of CYP2C19 Genotypic Differences in the Metabolism of Omeprazole and Rabeprazole on Intragastric pH. Aliment. Pharmacol. Ther..

[59] Boyle J.T. (2003). Acid Secretion From Birth to Adulthood. J. Pediatr. Gastroenterol. Nutr..

[60] Pippa L.F., Vozmediano V., Mitrov-Winkelmolen L., Touw D., Soliman A., Cristofoletti R., Salgado Junior W., de Moraes N.V. (2024). Impact of Obesity and Roux-En-Y Gastric Bypass on the Pharmacokinetics of (R)- and (S)-Omeprazole and Intragastric pH. CPT: Pharmacomet. Syst. Pharmacol..

[61] Kirchheiner J., Glatt S., Fuhr U., Klotz U., Meineke I., Seufferlein T., Brockmöller J. (2009). Relative Potency of Proton-Pump Inhibitors—Comparison of Effects on Intragastric pH. Eur. J. Clin. Pharmacol..

[62] Jonathan Bishop Ã., Furman M., Mike Thomson Ã Sheffield Children Ã. (2007). Omeprazole for Gastroesophageal Reflux Disease in the First 2 Years of Life: A Dose-Finding Study with Dual-Channel pH Monitoring. J. Pediatr. Gastroenterol. Nutr..

[63] Gan J., Bornhorst G.M., Henrick B.M., German J.B. (2018). Protein Digestion of Baby Foods: Study Approaches and Implications for Infant Health. Mol. Nutr. Food Res..

[64] Shah N., Gossman W. (2025). Omeprazole. StatPearls [Internet].

[65] Strand D.S., Kim D., Peura D.A. (2017). 25 Years of Proton Pump Inhibitors: A Comprehensive Review. Gut Liver.

[66] Rouaz-El Hajoui K., Chiclana-Rodríguez B., Nardi-Ricart A., Suñé-Pou M., Mercadé-Frutos D., María Suñé-Negre J., Pérez-Lozano P., García-Montoya E. (2023). Formulation of Omeprazole in the Pediatric Population: A Review. J. Pharm. Sci. Drug Discov..

[67] Katashima M., Yamamoto K., Tokuma Y., Hata T., Sawada Y., Iga T. (1998). Comparative Pharmacokinetic/Pharmacodynamic Analysis of Proton Pump Inhibitors Omeprazole, Lansoprazole and Pantoprazole, in Humans. Eur. J. Drug Metab. Pharmacokinet..

[68] Kaye J.L. (2011). Review of Paediatric Gastrointestinal Physiology Data Relevant to Oral Drug Delivery. Int. J. Clin. Pharm..

[69] Rødbro P., Krasilnikoff P.A., Christiansen P.M. (1967). Parietal Cell Secretory Function in Early Childhood. Scand. J. Gastroenterol..

[70] Wills L., Paterson D. (1926). A Study of Gastric Acidity in Infants. Arch. Dis. Child..

[71] Bartelink I.H., Rademaker C.M.A., Schobben A.F.A.M., Van Den Anker J.N. (2006). Guidelines on Paediatric Dosing on the Basis of Developmental Physiology and Pharmacokinetic Considerations. Clin. Pharmacokinet..

[72] Energy In: Recommended Food & Drink Amounts for Children. https://www.healthychildren.org/English/healthy-living/nutrition/Pages/Energy-In-Recommended-Food-Drink-Amounts-for-Children.aspx.

[73] Amount and Schedule of Baby Formula Feedings. https://www.healthychildren.org/English/ages-stages/baby/formula-feeding/Pages/amount-and-schedule-of-formula-feedings.aspx.

[74] Sample Menu for A Two-Year-Old. https://www.healthychildren.org/English/ages-stages/toddler/nutrition/Pages/Sample-One-Day-Menu-for-a-Two-Year-Old.aspx.

[75] Sample Menu for A Baby 8 to 12 Months Old. https://www.healthychildren.org/English/ages-stages/baby/feeding-nutrition/Pages/sample-one-day-menu-for-an-8-to-12-month-old.aspx.

[76] Online Food Calculator Food Weight to Volume Conversions. https://www.aqua-calc.com/calculate/food-weight-to-volume.

