# Supplementary material for: Leveraging Omeprazole PBPK/PD Modeling to Inform Drug–Drug Interactions and Specific Recommendations for Pediatric Labeling
Source: Pharmaceutics. 2025 Mar 14;17(3):373. doi: 10.3390/pharmaceutics17030373 (PMC11944414; doi:10.3390/pharmaceutics17030373)
Supplement: Supplementary file 1 [file pharmaceutics-17-00373-s001.zip › pharmaceutics-3408382-supplementary.pdf]

pharmaceutics

MDPI

Supplementary material

List of Tables and Figures

Table S1. Summary of pharmacodynamic model parameters, based on Liu et al. [44]

Table S2. Key physicochemical and biopharmaceutical parameters for metabolite Hydroxy-Omeprazole.

Table S3. Key physicochemical and biopharmaceutical parameters for metabolite Omeprazole Sulphone.

Table S4. Initial physiologically based pharmacokinetic (PBPK) Model - Simulated versus observed PK parameters for omeprazole intravenous and oral doses in children.

Table S5. DDI dynamic single simulation- Simulated PK and PD parameters and ratios for omeprazole administrated alone and as a victim with a hypothetical drug which fully blocks the CYP 3A enzymes as a perpetrator in healthy subjects.

Table S6. DDI dynamic population simulation (100 subjects)- Simulated PK and PD parameters and ratios for omeprazole administrated alone and as a victim with a hypothetical drug which fully blocks the CYP 3A enzymes as a perpetrator in healthy different ages populations.

Figure S1. PBPK model development; Predicted versus observed plasma concentrations obtained after intravenous administration of 40 mg of omeprazole (a), oral administration of 40 mg of omeprazole (b) and oral administration of 90 mg of omeprazole (c). Omeprazole (grey), hydroxy-omeprazole (blue) and omeprazole sulphone (orange). Observed data; Andersson et al, 1990 [27]

Figure S2. Verification of omeprazole PBPK model in adults after multiple dosing administration.

Figure S3. Predicted versus observed plasma concentration profiles obtained after oral administration of 16.7 mg of omeprazole in 6-year-old children with inclusion of autoinhibition and mechanism-based inactivation processes. Observed data; Marier et al. 2004 [28]

Figure S4. Verification of the contribution of CYP2C19 for omeprazole metabolism in omeprazole PBPK model in adults.

Figure S5. Single simulation results of different age subjects after the administration of omeprazole alone and in combination with CYP3A inhibition.

Tables

Table S1. Summary of pharmacodynamic model parameters, based on Liu et al. [1]

Parameter	Definition	Populations				
		37 year-old adults	6 year-old children	2 year-old children	8 months-old infants	4 months-old infants
k_{deg} (1/h)	Degradation rate constant of H^+/K^+-APTase			0.00827		
k_d (1/h/ng/mL)	Efficacy of omeprazole to irreversibly bind to H^+/K^+-APTase			0.00388 [67]		
$Base^{\phi}$ (mM)	Baseline of intra-gastric H^+ concentration	40.1 [44] (pH 1.4)	31.62 [68] (pH 1.5)	16.59 [69] (pH 1.78)	1.2 [70] (pH 2.92)	0.79 [70] (pH 3.1)
k_{out} (1/h)	Elimination rate constant for intra-gastric H^+ concentration			3.83		
MW (h)	Width of the night intragastric H^+ surge			0.0580		
MA (unit)	Amplitude of the night intragastric H^+ surge			537		
MT_{max} (h)	Peak time of the night intragastric H^+ surge			20.1		
V_{ef} #	Effect of stomach volume	1	0.1036	0.04866	0.0375	0.0352
V_{ef1} *(mL)	Food effect of meal no. 1 (breakfast)	540	410	290	330	
V_{ef2} *(mL)	Food effect of meal no. 2 (snack)	270	180	120	240	
V_{ef3} *(mL)	Food effect of meal no. 3 (lunch)	600	440	360	330	
V_{ef5} * (mL)	Food effect of meal no. 5 (dinner)	630	380	330	510	
V_{efmilk} *(mL)	Food effect of breastmilk or formula	—	—	—	150	120
k_{fe} (1/h)	Elimination rate constant for food effect			0.694		
t_{lag} (h)	Lag time between plasma concentration and changes of intra-gastric H^+ concentration			0.396		

ϕ For pediatric population, *BASE* was updated from the literature to reflect the gradual decline in the intragastric pH during the early years of life until it reaches adult values [71].

# The built-in Gastroplus™ gastric volumes at fasting were 50, 5.18, 2.433, 1.875 and 1.76 mL for adults and for children aged 6 years, 2 years, 8 months, and 4 months, respectively.

* To assess the impact of food on intragastric acid concentration in real-life scenarios, samples of typical meals tailored to different age groups were utilized [72–75] . Different food meals contents were converted to volumes using food calculator [76].

Table S2. Key physicochemical and biopharmaceutical parameters for metabolite Hydroxy-Omeprazole.

Parameter	Value	Reference
logP	0.86	ADMET Predictor v.9.5
Diffusion coefficient	0.7 x 10^{-5} cm^2/s	ADMET Predictor v.9.5
pKa	11.19 (acid)<br>4.23 (Base)<br>2.55 (Base)	ADMET Predictor v.9.5
Reference solubility	1.15 mg/mL a pH = 7.63	ADMET Predictor v.9.5
Solubility Factor	66.07	ADMET Predictor v.9.5
FaSSIF solubility	0.41 mg/mL a pH = 6.5	ADMET Predictor v.9.5
FeSSIF solubility	0.54 mg/mL a pH = 5.0	ADMET Predictor v.9.5
Human effective permeability (P_{eff})	1.02 x 10^{-4} cm/s	ADMET Predictor v.9.5
Drug particle density	1.2 g/mL	GastroPlus v.9.8 default
Mean precipitation time	900 s	GastroPlus v.9.8 default
Blood:plasma concentration ratio (R_{bp})	0.6	ADMET Predictor v.9.5
Plasma protein binding (Fup%)	23.3 %	ADMET Predictor v.9.5
Final adjusted Fup%	4.989 %	Fitted
**Metabolism**		
**Degradation of Hydroxy Omeprazole**		
Lumped MP $K_{m,u}$ (PBPK)	0.16 mg/L	[37]
Lumped MP V_{max} (PBPK)	7×10^{-7} mg/s/mg enzyme	Initially informed from invitro [37] then fitted
CYP3A4 $K_{m,u}$ (PBPK)	24.91 mg/L	[37]
CYP3A4 V_{max} (PBPK)	9.47 ×10^{-7} mg/s/mg enzyme	Initially informed from invitro [37] then fitted
CYP3A7 $K_{m,u}$ (PBPK)	123.44 mg/L	Calculated from Km for CYP3A4 [41]
CYP3A7 V_{max} (PBPK)	2.37 ×10^{-7} mg/s/mg enzyme	Calculated from Vmax for CYP3A4 [41]

Table S3. Key physicochemical and biopharmaceutical parameters for metabolite Omeprazole Sulphone.

Parameter	Value	Reference
logP	2.24	ADMET Predictor v.9.5
Diffusion coefficient	0.69×10^{-5} cm^2/s	ADMET Predictor v.9.5
pKa	10.89 (acid)<br>3.93 (base)<br>1.79 (base)	ADMET Predictor v.9.5
Reference solubility	3.09 mg/mL a pH =3.15	ADMET Predictor v.9.5
Solubility Factor	449.9	ADMET Predictor v.9.5
FaSSIF solubility	0.043 mg/mL a pH = 6.5	ADMET Predictor v.9.5
FeSSIF solubility	0.15 mg/mL a pH = 5.0	ADMET Predictor v.9.5
Human effective permeability (P_{eff})	0.67×10^{-4} cm/s	ADMET Predictor v.9.5
Drug particle density	1.2 g/mL	GastroPlus v.9.8 default
Mean precipitation time	900 s	GastroPlus v.9.8 default
Blood:plasma concentration ratio (R_{bp})	0.6	ADMET Predictor v.9.5
Plasma protein binding (Fup%)	11.3 %	ADMET Predictor v.9.5
Final adjusted Fup%	4.749 %	Fitted
**Metabolism**		
**Degradation of Omeprazole Sulphone**		
CYP2C19 $K_{m,u}$ (PBPK)	0.058 mg/L	[37]
CYP2C19 V_{max} (PBPK)	5.17×10^{-5} mg/s/mg enzyme	Initially informed from invitro [37] then fitted
CYP3A4 $K_{m,u}$ (PBPK)	8.46 mg/mL	[37]
CYP3A4 V_{max} (PBPK)	9.89×10^{-4} mg/s/mg enzyme	Initially informed from invitro [37] then fitted
CYP3A7 $K_{m,u}$ (PBPK)	42.966 mg/mL	Calculated from Km for CYP3A4 [41]
CYP3A7 V_{max} (PBPK)	2.47×10^{-4} mg/s/mg enzyme	Calculated from Vmax for CYP3A4 [41]

Table S4. Initial physiologically based pharmacokinetic (PBPK) Model - Simulated versus observed PK parameters for omeprazole intravenous and oral doses in children.

Dose [mg]	Route	Age [years]	Weight [kg]	$AUC_{0-\infty}$ (Sim.) [µg·h/mL]	$AUC_{0-\infty}$ (Obs.) [µg·h/mL]	$AUC_{0-\infty}$ ratio (Sim./Obs.)	Reference
3.78	1-hour iv infusion once daily	0.5	6.2	2.99	1.64	1.82	[31]
5.57	1-hour iv infusion once daily	1.8	10.5	1.1	0.71	1.55	[31]
6.14	1-hour iv infusion once daily	1.3	11.8	1.6	0.85	1.88	[31]
5.60	1-hour iv infusion once daily	2.3	10	1.12	0.55	2.04	[31]
7.98	1-hour iv infusion once daily	0.8	7	5.39	1.43	3.77	[31]
8.31	1-hour iv infusion once daily	1.1	7.1	4.81	1.90	2.53	[31]
8.97	1-hour iv infusion once daily	1.3	7.8	4.73	4.9	0.97	[31]
10.36	1-hour iv infusion once daily	1.3	9.5	4.45	3.78	1.18	[31]
7.02	1-hour iv infusion once daily	0.4	5.4	8.7	7.71	1.13	[31]
20	Oral	1.6	11.6	4.44	2.00	2.22	[32]
20	Oral	4.2	15.7	2.67	0.38	7.03	[32]
30	Oral	5	13.8	4.49	3.80	1.18	[32]
20	Oral	5.1	16.2	2.28	2.52	0.90	[32]
15	Oral	6.1	24.5	1.09	0.55	1.99	[32]
50	Oral	7	18.2	9.19	6.90	1.33	[32]
17.5	Oral	8.4	27	1.28	1.24	1.03	[32]
30	Oral	9.6	43.4	1.77	3.14	0.56	[32]
25	Oral	10.7	39	1.40	1.62	0.87	[32]
45	Oral	11.2	68	3.22	2.86	1.13	[32]
17.5	Oral	12.6	39	0.81	0.90	0.89	[32]
32.5	Oral	12.7	49	1.61	2.59	0.62	[32]
80	Oral	15	27.6	9.52	7.59	1.25	[32]
80	Oral	15.3	52.5	5.55	5.52	1.01	[32]
75	Oral	15.3	72.2	3.78	7.59	0.50	[32]
80	Oral	16.1	63.2	4.60	7.25	0.63	[32]
50	Oral	16.2	66.5	2.17	3.42	0.63	[32]

Colors are to know which ones are within 1.25-fold (green), 2-fold (yellow) and those who did not fit the limits (red).

Table S5. DDI dynamic single simulation- Simulated PK and PD parameters and ratios for omeprazole administrated alone and as a victim with a hypothetical drug which fully blocks the CYP 3A enzymes as a perpetrator in healthy subjects.

Dose [mg]	Route	Age	Weight [kg]	Parameter	(OMP) [μg·h/mL]	(OMP+ CYP3A inhibitor) [μg·h/mL]	Ratio (OMP+ CYP3A inhibitor/OMP)	Reference
6	Oral	4 months	4.8	**$AUC_{336–360}$**	7.78	24.86	3.19	[30]
				**$AUEC_{336–360}$**	1.65	1.69	1.03	
8	Oral	8 months	6.97	**$AUC_{336–360}$**	5.36	20.45	3.82	[30]
				**$AUEC_{336–360}$**	1.77	1.83	1.04	
10	Oral	2 years	12.87	**$AUC_{336–360}$**	1.52	3.89	2.56	[28]
				**$AUEC_{336–360}$**	1.91	1.99	1.04	
16.7	Oral	6 years	24.2	**$AUC_{336–360}$**	1.28	2.74	2.13	[28]
				**$AUEC_{336–360}$**	2.49	2.75	1.10	
20	Oral	37 years	71	**$AUC_{336–360}$**	0.54	0.79	1.47	[28]
				**$AUEC_{336–360}$**	2.54	2.83	1.12	

$AUC_{336–360}$: area under the curve from 336-360 hr; **$AUEC_{336–360}$**: area under the effective curve from 336–360 hr, Week DDI, moderate DDI, OMP: omeprazole.

Table S6. DDI dynamic population simulation (100 subjects)- Simulated PK and PD parameters and ratios for omeprazole administrated alone and as a victim with a hypothetical drug which fully blocks the CYP 3A enzymes as a perpetrator in healthy different ages populations.

**Route**	**Population Age**	**Gender % male**	**Weight [kg]** Mean ± SD	**Parameter**	**(OMP)** Median (5th and 95th percentiles of the geometric means)	**(OMP+ full CYP3A inhibition)** Median (5th and 95th percentiles of the geometric means)	**Ratio (OMP+ CYP3A inhibition/OMP)**
Oral	4 months	52	6.6 ± 0.53	**$AUC_{186-192}$ [ng.h/mL]**	2900.83 (2270.74–2994.01)	8604.55 (6584.76–9467.65)	3.06 (2.71–3.39)
				**C_{max} [ng.h/mL]**	1137.79 (1001.27–1144.12)	1564 (1349.05–1537.26)	1.29 (1.29–1.39)
				**$AUEC_{186-192}$**	140.29 (139-141.15)	168.79 (166.51-170.81)	1.21 (1.18-1.22)
Oral	8 months	43	9.11 ± 0.73	**$AUC_{186-192}$ [ng.h/mL]**	1998.78 (1711.71–2326.14)	8314.12 (4864.08-7389.28)	2.96 (2.64–3.42)
				**C_{max} [ng.h/mL]**	1092.84 (934.38–1091.41)	1489.58 (1263.65–1474.52)	1.27 (1.29–1.41)
				**$AUEC_{186-192}$**	146.6 (146.2-148.55)	161.98 (157.28-161.74)	1.08 (1.07-1.09)
Oral	2 years	55	12.4 ± 1.01	**$AUC_{186-192}$ [ng.h/mL]**	872.75 (815.62– 1117.25)	2892.43 (2082.36–3341.51)	2.47 (2.37–3.22)
				**C_{max} [ng.h/mL]**	709.77 (609.29–741.93)	1099.09 (879.06–1092.85)	1.33 (1.37–1.55)
				**$AUEC_{186-192}$**	109.48 (108.7-110.88)	117.33 (116.43-120.70)	1.06 (1.07-1.09)
Oral	6 years	49	23.5 ± 1.87	**$AUC_{186-192}$ [ng.h/mL]**	1147.17 (903.71–1201.663)	2812.49 (2032.13–3212.25)	2.09 (2.13–2.81)
				**C_{max} [ng.h/mL]**	730.94 (632.40–768.83)	1109.55 (862.33–1077.52)	1.29 (1.31–1.45)
				**$AUEC_{186-192}$**	107.89 (106.22-108.41)	109.73 (108.18-110.96)	1.01 (1.02–1.03)
Oral	37 years	51	85.27±8.45	**$AUC_{186-192}$ [ng.h/mL]**	603.78 (493.13–657.4)	768.92 (632.15–885.41)	1.24 (1.26–1.37)
				**C_{max} [ng.h/mL]**	248.66 (209.34–266.94)	281.18 (244.53–315.72)	1.15 (1.15–1.20)
				**$AUEC_{186-192}$**	103.39 (101.43-103.65)	105.23 (103.28- 105.88)	1.01 (1.01-1.02)

The simulated omeprazole doses were 20 mg for adults and 0.7mg/kg for pediatrics, administrated once daily for 8 days.
$AUC_{186-192}$: area under the curve from 186 to 192 h (day8); **$AUEC_{186-192}$**: area under the effective curve from 186 to 192 hr, Week DDI, moderate DDI, OMP: omeprazole.

Figures

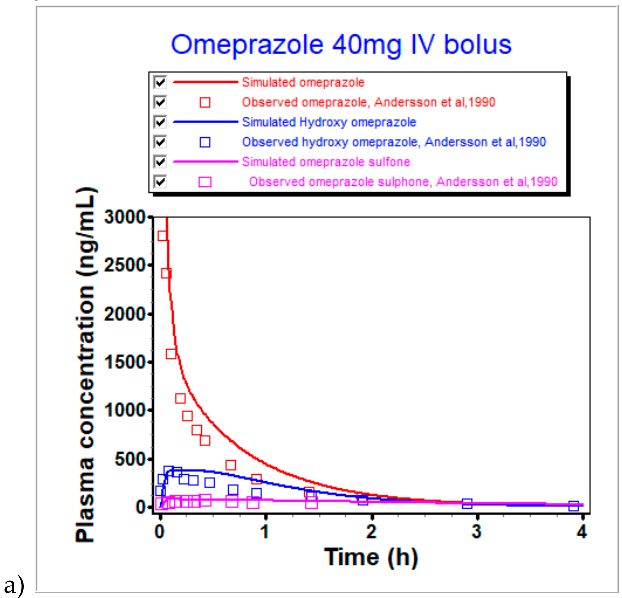

a)

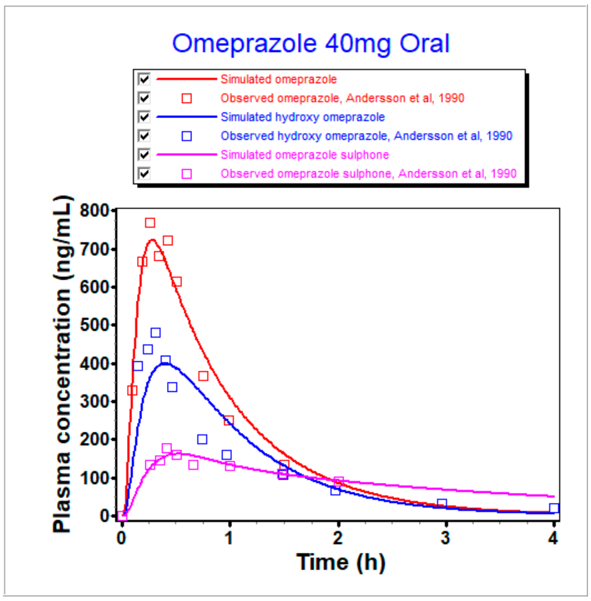

b)

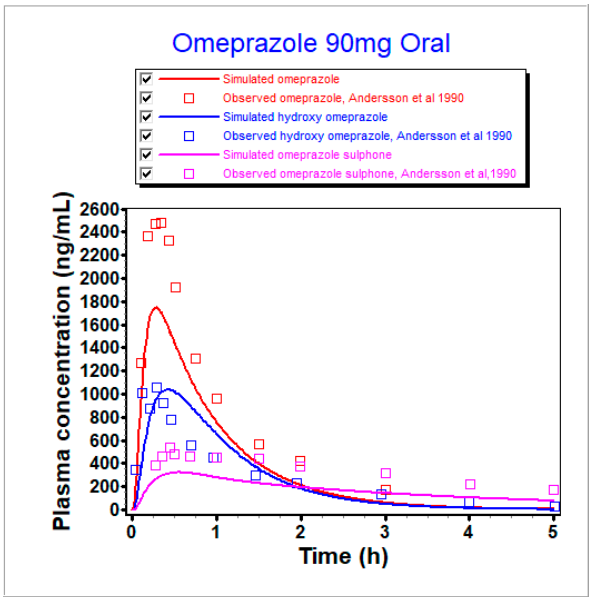

c)

Figure S1. PBPK model development; Predicted versus observed plasma concentrations obtained after intravenous administration of 40 mg of omeprazole (a), oral administration of 40 mg of omeprazole (b) and oral administration of 90 mg of omeprazole (c). Omeprazole (grey), hydroxy-omeprazole (blue) and omeprazole sulphone (orange). Observed data; Andersson et al, 1990 [27]

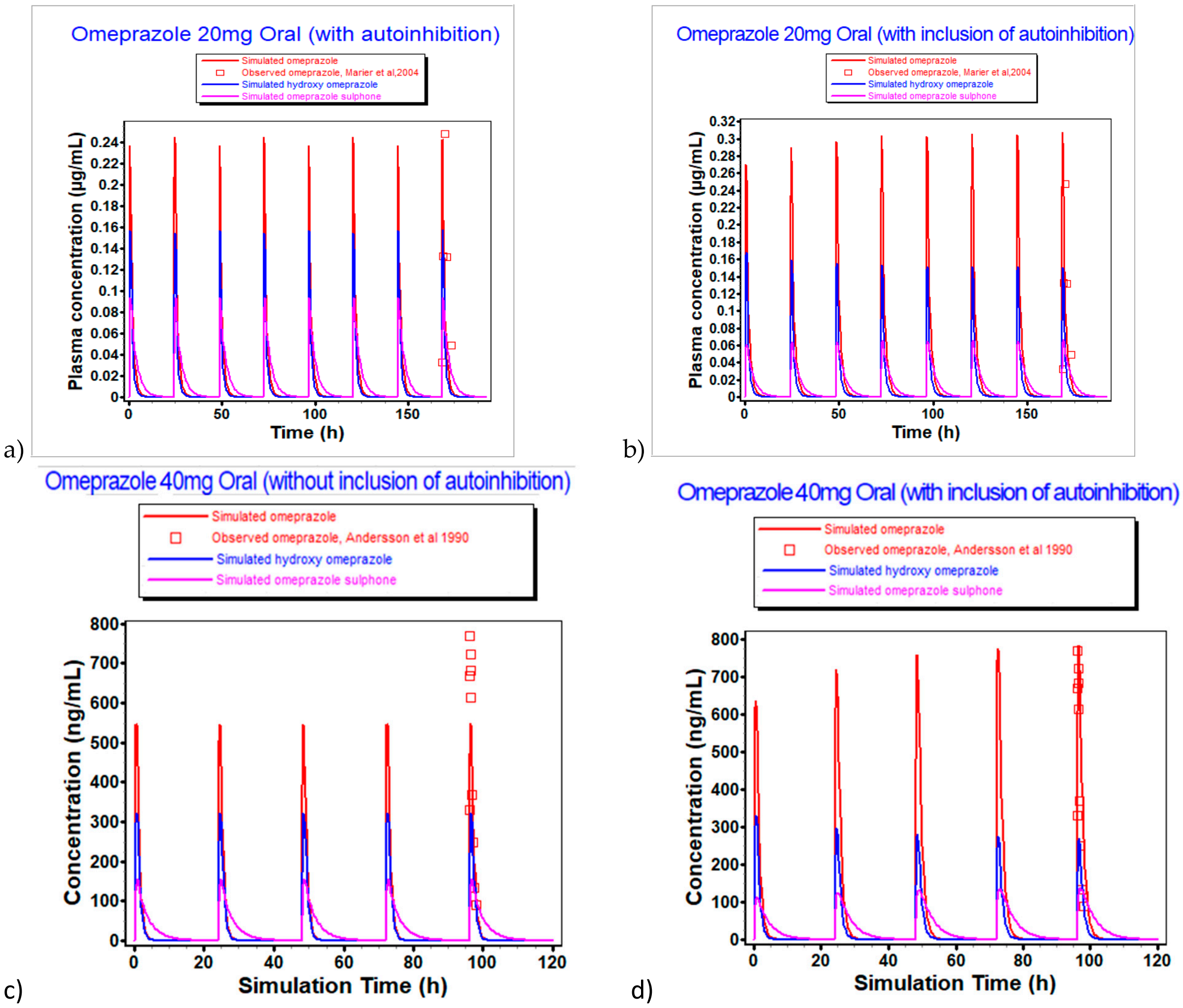

Figure S2. Validation of omeprazole PBPK model in adults after multiple dosing administration.

Predicted versus observed plasma concentration profiles obtained after multiple daily oral administration: 20 mg of delayed release omeprazole tablet for 8 days (a) without and (b) with inclusion of autoinhibition and mechanism-based inactivation mechanisms. Observed data; Marier et al. 2004 [28]
40 mg of omeprazole enteric coated granules for 5 days (c) without and (d) with inclusion of autoinhibition and mechanism-based inactivation mechanisms. Observed data; Andersson et al, 1991 [29]
Omeprazole (grey), hydroxy-omeprazole (blue) and omeprazole sulphone (orange).

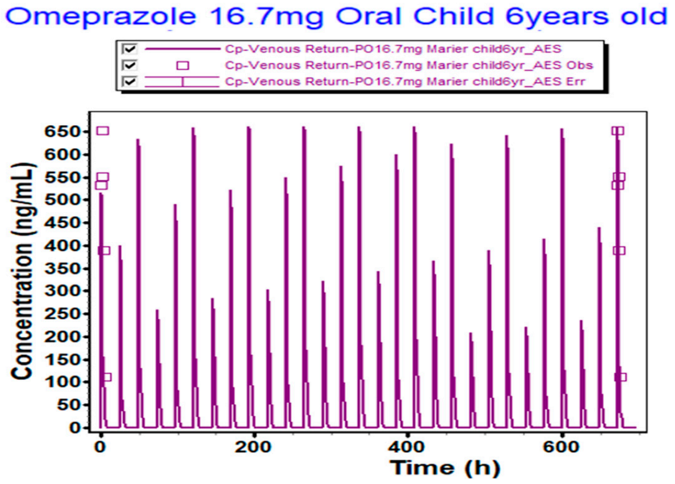

Figure S3. Predicted versus observed plasma concentration profiles obtained after oral administration of 16.7 mg of omeprazole in 6-year-old children with inclusion of autoinhibition and mechanism-based inactivation processes. Observed data; Marier et al. 2004 [28]

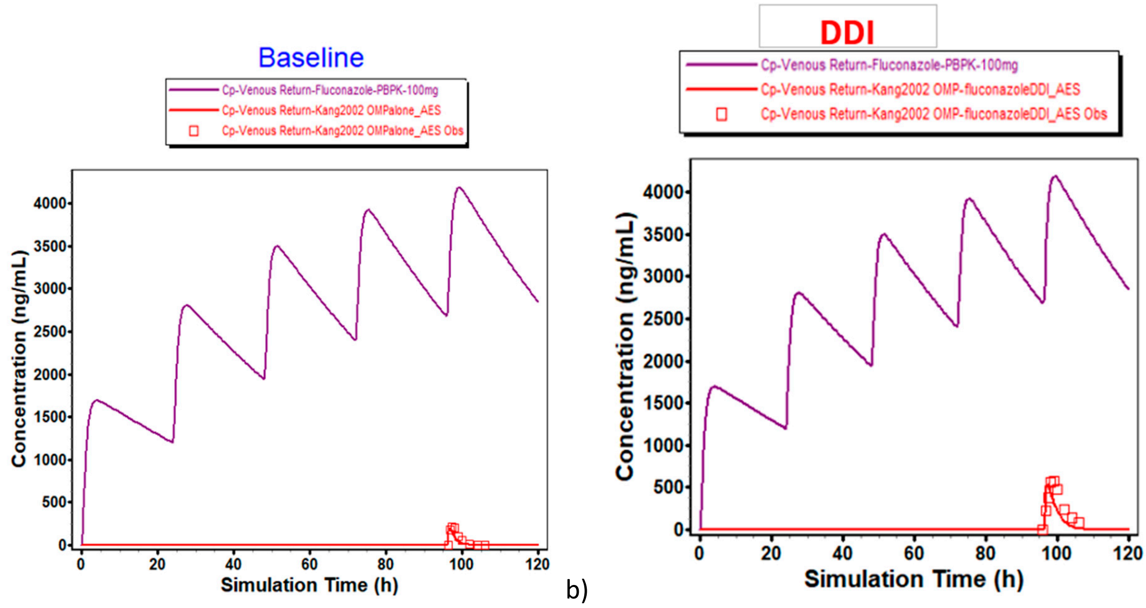

Figure S4. Verification of the contribution of CYP2C19 for omeprazole metabolism in omeprazole PBPK model in adults. a) simulation of baseline omeprazole 20mg without interaction with fluconazole (strong CYP2C19 and 2C9 inhibitor). b) simulation of omeprazole 20mg interaction with 100mg fluconazole (strong CYP2C19 and 2C9 inhibitor) at day5. Observed data: Kang et al,2002 [38]

(a)

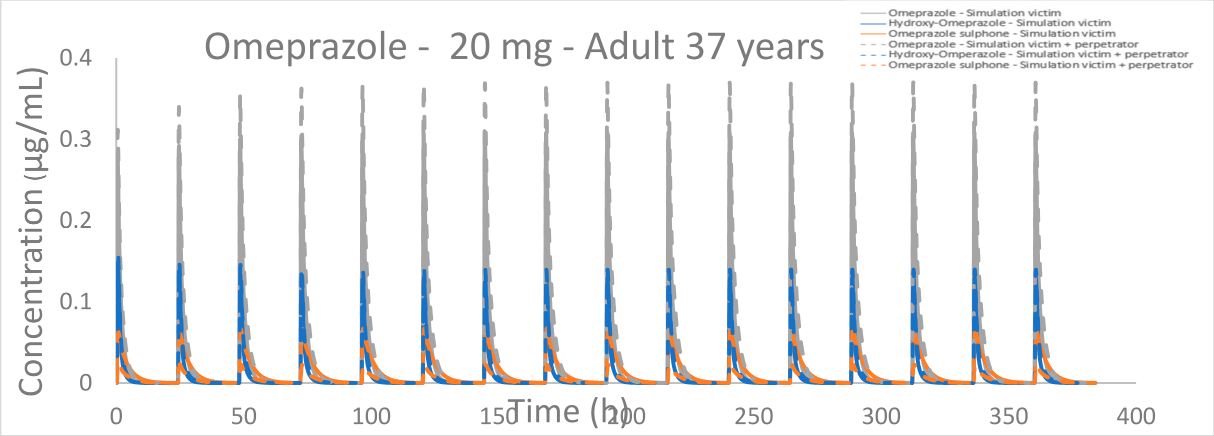
Omeprazole - 20 mg - Adult 37 years
Omeprazole - Simulation victim
Hydroxy-Omeprazole - Simulation victim
Omeprazole sulphone - Simulation victim
Omeprazole - Simulation victim + perpetrator
Hydroxy-Omperazole - Simulation victim + perpetrator
Omeprazole sulphone - Simulation victim + perpetrator
Concentration (µg/mL)
0.4
0.3
0.2
0.1
0
0
50
100
150
200
250
300
350
400
Time (h)

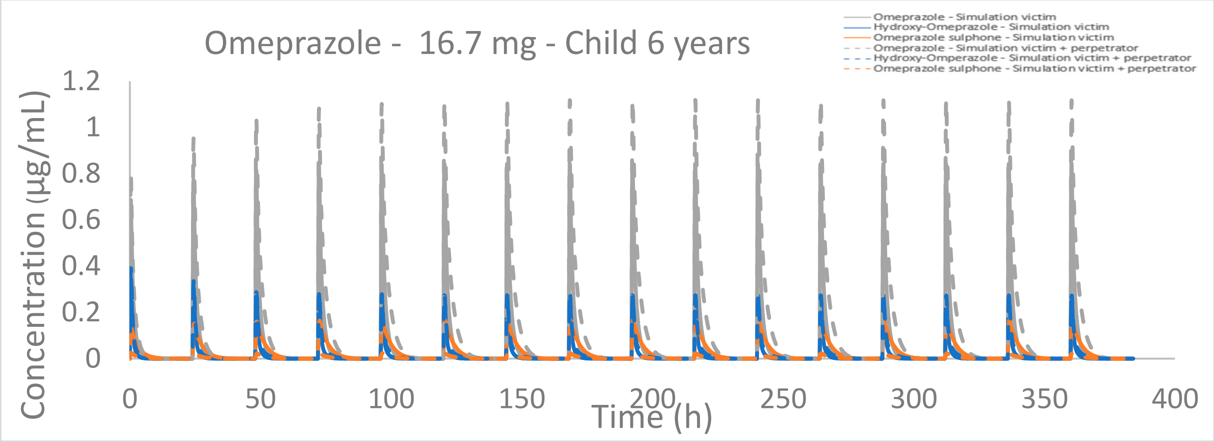
Omeprazole - 16.7 mg - Child 6 years
Omeprazole - Simulation victim
Hydroxy-Omeprazole - Simulation victim
Omeprazole sulphone - Simulation victim
Omeprazole - Simulation victim + perpetrator
Hydroxy-Omperazole - Simulation victim + perpetrator
Omeprazole sulphone - Simulation victim + perpetrator
Concentration (µg/mL)
1.2
1
0.8
0.6
0.4
0.2
0
0
50
100
150
200
250
300
350
400
Time (h)

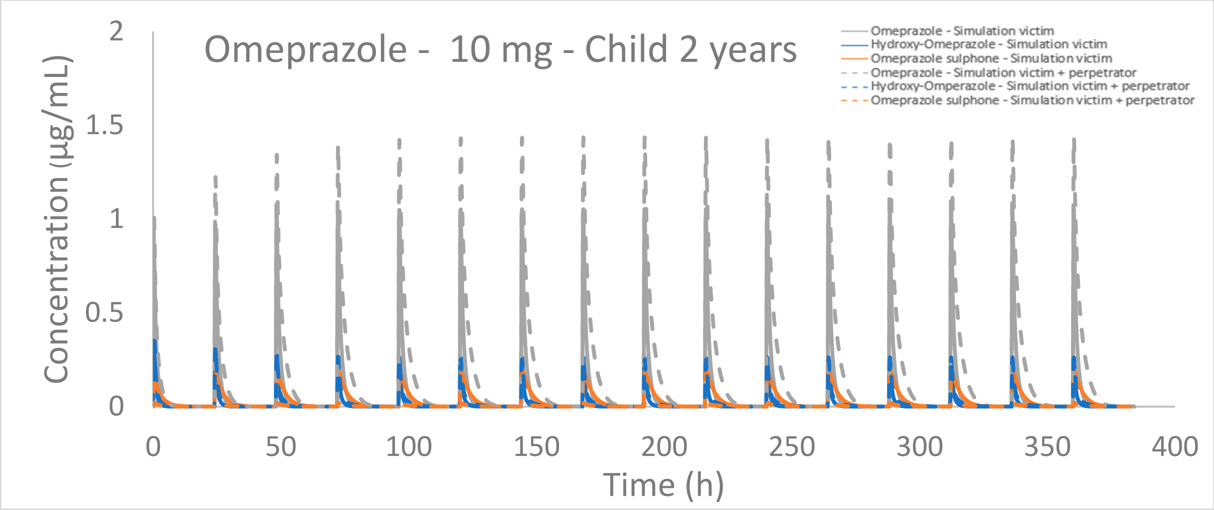
Omeprazole - 10 mg - Child 2 years
Omeprazole - Simulation victim
Hydroxy-Omeprazole - Simulation victim
Omeprazole sulphone - Simulation victim
Omeprazole - Simulation victim + perpetrator
Hydroxy-Omperazole - Simulation victim + perpetrator
Omeprazole sulphone - Simulation victim + perpetrator
Concentration (µg/mL)
2
1.5
1
0.5
0
0
50
100
150
200
250
300
350
400
Time (h)

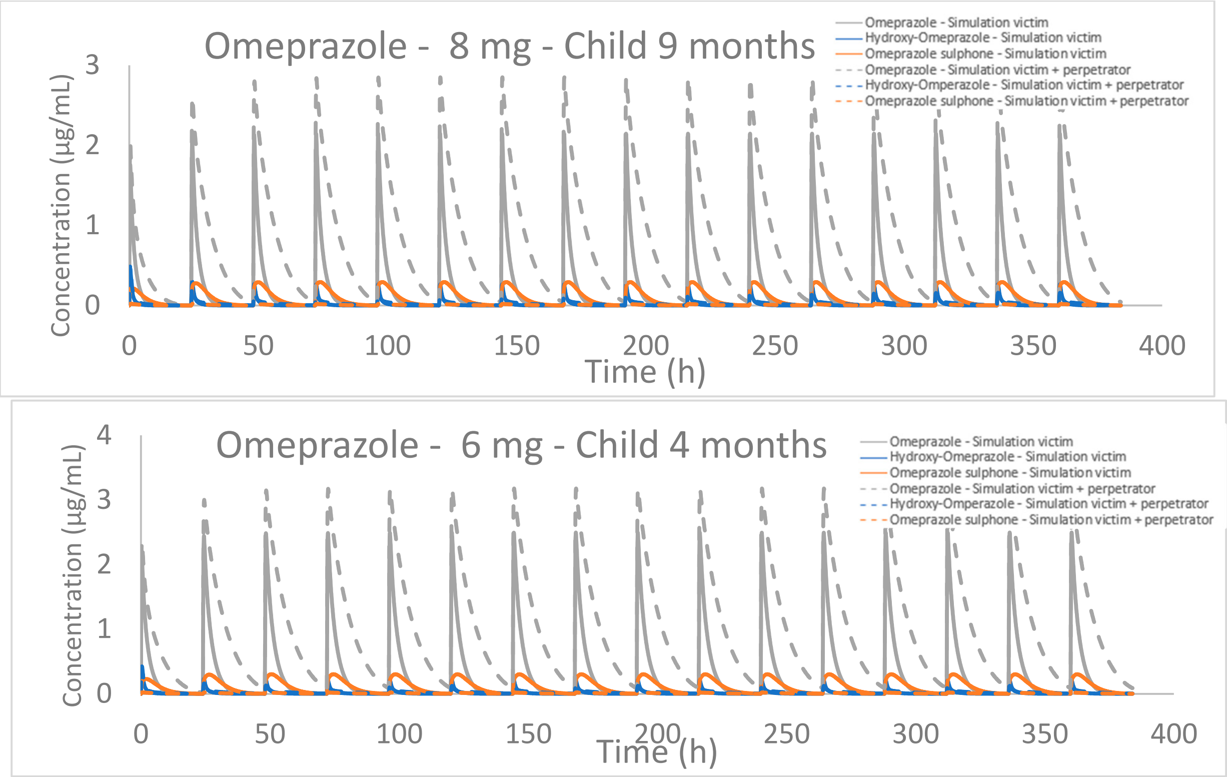

(b)

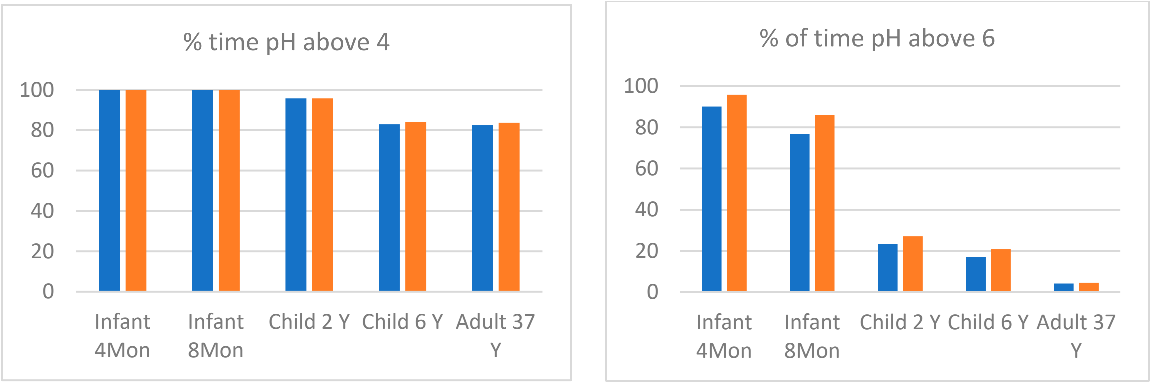

Figure S5. Single simulation results of different age subjects after the administration of omeprazole alone and in combination with CYP3A inhibition.

(a) Simulated plasma omeprazole concentrations in a subject aged 4 months, 8 months, 2 years, 6 years, and 37 years on day 8 after the administration of 6 mg, 8 mg,10 mg, 16.7 mg, and 20 mg, respectively of omeprazole alone and in combination with CYP3A inhibition.

Concentration-time profiles of omeprazole (grey), hydroxy-omeprazole (blue), and omeprazole sulphone (orange) after the administration of omeprazole alone (solid line) and in combination with CYP3A inhibition (dashed line).

(b) Percentage daily intragastric pH above 4 (right panel) and 6 (left panel) in a subject aged 4 months, 8 months, 2 years, 6 years, and 37 years on day 8 after the administration of 6 mg, 8 mg,10 mg, 16.7 mg, and 20 mg, respectively of omeprazole alone (blue) and in combination with CYP3A inhibition (orange).

Semi-mechanistic model to predict intragastric pH

A mechanism-based PD model[44] that integrates the drug effect, as well as the circadian rhythm and food effect on intragastric pH, was implemented in Pumas. Age-based physiological changes in gastric pH, intragastric volumes, and food effect were also implemented into the model. Simulations were carried out to assess the impact of age-based physiological changes in the GI on drug response in adults and pediatrics of different age after omeprazole administration. Intragastric H^+ secretion was used as a surrogate for drug response at different age populations.

The response model was previously established and verified by Liu *et al.* [44] in healthy volunteers with accurate predictions of intragastric pH over 24 hours accounting for the irreversible inhibition of H^+/K^+-ATPase, a food dilution effect in the intragastric concentration of H^+ and the circadian rhythm which leads to an increase in gastric pH during nighttime. The zero-order secretion rate constants of gastric H^+ at 0 ($k_{in(0)}$) and t hours ($k_{in(t)}$) accounted for an asymmetric circadian effect on acid secretion (Equations S1 and S2).

$$k_{in(0)} = k_{out} \times BASE \times \left(1 + \frac{MA}{\left(\frac{MT_{max}}{MW}\right)^4 + 1}\right) \quad \text{(S1)}$$

$$k_{in(t)} = \frac{k_{in(0)}}{1 + \frac{MA}{\left(\frac{T - MT_{max}}{MW}\right)^4 + 1}} \quad \text{(S2)}$$

The first-order elimination rate constant of intragastric H^+ concentration (k_{out}) was 3.83 1/h [44]. *BASE* is the baseline intragastric H^+ concentration, set at 40.1 mM (pH 1.4) for adult patients, as documented by Liu *et al.* [44]. However, *BASE* was updated from the literature for the pediatric population to reflect the gradual decline in the intragastric pH during the first years of life until it reaches adult values [71]. *BASE* values were set at 31.62 mM (pH 1.5) [68], 16.59 mM (pH 1.78) [69], 1.2 mM (pH 2.92) [70], and 0.79 mM (pH 3.1) [70] for children aged 6 years, 2 years, 8 months

and 4 months, respectively. *MA*, *MW*, and MT_{max} are used to describe the amplitude, width, and mean time of the circadian rhythm effect on gastric H^+ secretion with values set as 398 (unit), 1.25 h and 22.4 h, respectively.

The built-in GastroPlus parameters were employed to gather intragastric volume values for each age group. To accommodate variations in intragastric volumes between adults and pediatric individuals of different ages, the intragastric volumes for children of different ages were incorporated into the model as a ratio relative to adults, where the stomach volume ratio (V_{Ef}) was set to 1 for adults. Specifically, the V_{Ef} was adjusted to 0.1036, 0.04866, 0.0375, and 0.0352 for children aged 6 years, 2 years, 8 months, and 4 months, respectively, as shown in Table S1.

Samples of typical meals tailored to different age groups were utilized [9–12]. Different meal contents were converted to volumes using a food calculator [76] to investigate the impact of food on intragastric acid concentration under real-life situations. The daily food effect was figured based on three main meals (breakfast, lunch, and dinner) and two snacks between each main meal ($V_{Ef1\text{–}5}$) at specific time intervals (1, 3, 5, 8, and 11 h) following drug administration across all ages, excluding infants under one year of age (Equation S3). For infants at 8 months of age, a similar approach was adopted to that of older age groups, with the addition of a bedtime feeding of breastmilk or formula ($V_{Ef\text{milk}}$=150 mL) at 13 h post-drug administration (Equation S4). In the case of 4-month-old infants, the food effect was implemented to simulate the intake of 6 repeated breastmilk or formula of equal volumes ($V_{Ef\text{milk}}$=120 mL) every four hours over 24 h where the first intake at 1 h after drug administration (Equation S5). The volumes of food for main meals, breastmilk, or formula were standardized to be administered within a 30-minute window, while snacks were evaluated as boluses. The elimination rate constant for the food effect (k_{fe}) was set at 0.694 1/h, reflecting gastric emptying. Estimated food volumes for each age population is detailed in Table S1.

$$\frac{dV_{Ef}}{dt} = \frac{V_{Ef1}}{0.5}(\text{if } t \text{ is within } 1.0\text{~}1.5) + \frac{V_{Ef2}}{0.5}(\text{if } t = 3) + \frac{V_{Ef3}}{0.5}(\text{if } t \text{ is within } 5.0\text{~}5.5) + \frac{V_{Ef4}}{0.5}(\text{if } t = 8) + \frac{V_{Ef5}}{0.5}(\text{if } t \text{ is within } 11.0\text{~}11.5) + k_{fe} - k_{fe} \times V_{Ef} \quad \text{(S3)}$$

$$\frac{dV_{Ef}}{dt} = \frac{V_{Ef1}}{0.5}(\text{if } t \text{ is within } 1.0\text{\textasciitilde}1.5) + \frac{V_{Ef2}}{0.5}(\text{if } t = 3)$$
$$+\frac{V_{Ef3}}{0.5}(\text{if } t \text{ is within } 5.0\text{\textasciitilde}5.5) + \frac{V_{Ef4}}{0.5}(\text{if } t = 8)$$
$$+\frac{V_{Ef5}}{0.5}(\text{if } t \text{ is within } 11.0\text{\textasciitilde}11.5) + \frac{V_{Efmilk}}{0.5}(\text{if } t \text{ is within } 13.0\text{\textasciitilde}13.5)$$
$$+ k_{fe} - k_{fe} \times V_{Ef} \qquad \text{(S4)}$$

$$\frac{dV_{Ef}}{dt} = \frac{V_{Efmilk}}{0.5}\begin{pmatrix} \text{if } t \text{ is within } 1.0\text{\textasciitilde}1.5 \\ \text{or within } 5.0\text{\textasciitilde}5.5 \\ \text{or within } 9.0\text{\textasciitilde}9.5 \\ \text{or within } 13.0\text{\textasciitilde}13.5 \\ \text{or within } 17.0\text{\textasciitilde}17.5 \\ \text{or within } 21.0\text{\textasciitilde}21.5 \end{pmatrix} \qquad \text{(S5)}$$

The relative baseline H^+/K^+-ATPase activity (E/E_0) was predicted as a function of the production or elimination rate constant of E/E_0 (k_{deg}), the potency of omeprazole in inhibiting H^+/K^+-ATPase activity (k_d) and plasma concentrations of omeprazole (Cp) (Equation S6). The plasma concentration predicted by our PBPK model with and without the DDI with a hypothetical CYP3A4 strong inhibition was used to drive impact of the PD in both adults and pediatrics of different ages.

The indirect irreversible response (IDR) model was employed to simulate the dynamics of intragastric H^+ concentration (Equation S7) after incorporating changes in the circadian rhythm of intragastric H^+ secretion ($k_{in(t)}$) and the influence of H^+/K^+-ATPase activity. The final observed intragastric H^+ concentration (H_{obs}) can be calculated considering the effect of food using Equation S8. An upper limit of 7.4 (physiological fluid pH 7.4) for the intragastric pH value was set. If H_{obs} was estimated to be lower than $10^{-7.4}$M, then it was set at $10^{-7.4}$M. Additional model parameters are displayed in Table S1.

$$\frac{d\,{}^{E}/_{E_0}}{dt} = k_{deg} - k_{deg} \times \frac{E}{E_0} - k_d \times \frac{E}{E_0} \times C_p \quad \text{(S6)}$$

$$\frac{dH}{dt} = k_{in(t)} \times \frac{E}{E_0} - k_{out} \times H \quad \text{(S7)}$$

$$H_{obs} = {}^{H}/_{V_{Ef}} \quad \text{(S8)}$$

Where k_{deg} was set as 0.00827 1/h [44]. The k_d of 0.00388 1/h/(ng/mL) was used account for the potencies of omeprazole in the inhibition of H^+/K^+-ATPase as reported by Katashima et al [67]. A lag time (t_{lag}) of 0.396 h was added to reflect the delay between plasma concentrations and changes of intragastric H^+ concentration [44].
